# Redox Polymers in Electrochemical Biosensing: Molecular Design, Electron Transfer, and Hybrid Nanocomposites

**DOI:** 10.3390/bios16090462

**Published:** 2026-08-25

**Authors:** Lyubov S. Kuznetsova, Kristina D. Ivanova, Jun Zhang, Vyacheslav A. Arlyapov

**Affiliations:** 1 Research Center “BioChemTech”, Tula State University, Lenin Avenue, 92, Tula 300012, Russia; 2 National Key Laboratory of Urban and Rural Water Resources and Water Environment, School of Environment, Harbin Institute of Technology, Harbin 150090, China; 3 Department of Chemistry, Tula State University, Lenin Avenue, 92, Tula 300012, Russia

**Keywords:** redox polymers, mediators, biosensors, electrochemical sensors, electronic transport, immobilization, composite materials

## Abstract

Redox-active polymers have become an important component of second-generation electrochemical biosensors, solving the problem of efficient charge transfer between the biological recognition material and the electrode surface. In this review, we discuss the basic design principles, electron transfer mechanisms, and synthesis strategies from the perspective of biosensor applications. Three main classes of redox centers are considered—metal complexes, metallocenes, and organic radicals—as well as polymer matrices, and the factors affecting their stability and operability are discussed. Particular attention is paid to hybrid nanocomposites based on carbon nanotubes, graphene, and metal nanoparticles. The review concludes that despite significant advances in molecular design and the development of nanocomposites, the commercialization of biosensors based on redox polymers is hindered by unresolved issues related to biofouling, metal center instability, and low reproducibility. This emphasizes the need for standardized synthesis and integration of machine learning-based design to achieve a balance between electron transfer kinetics, biocompatibility, and operational properties.

## 1. Introduction

Biosensors have taken a key place in modern analytical chemistry, reducing the cost of a single analysis and providing a high degree of automation. Biosensors are widely used in clinical diagnostics, environmental monitoring, food quality control, and biological safety [1,2,3]. Among them, electrochemical biosensors are of particular interest due to their combination of high sensitivity and portability [4,5,6].

Enzyme-based biosensors are the most common due to their high specificity to the substrate; however, the main limitation in their development is the low efficiency of charge transfer between the active center of the enzyme and the surface of the electrode. Due to the fact that the active center of enzymes is located deep inside the protein globule, charge transfer to the electrode is often difficult [7,8,9].

Initially, this problem was solved using dissolved mediators; however, this approach has significant drawbacks related to increased reagent consumption and the impossibility of creating portable analyzers [10].

**Figure 1:**
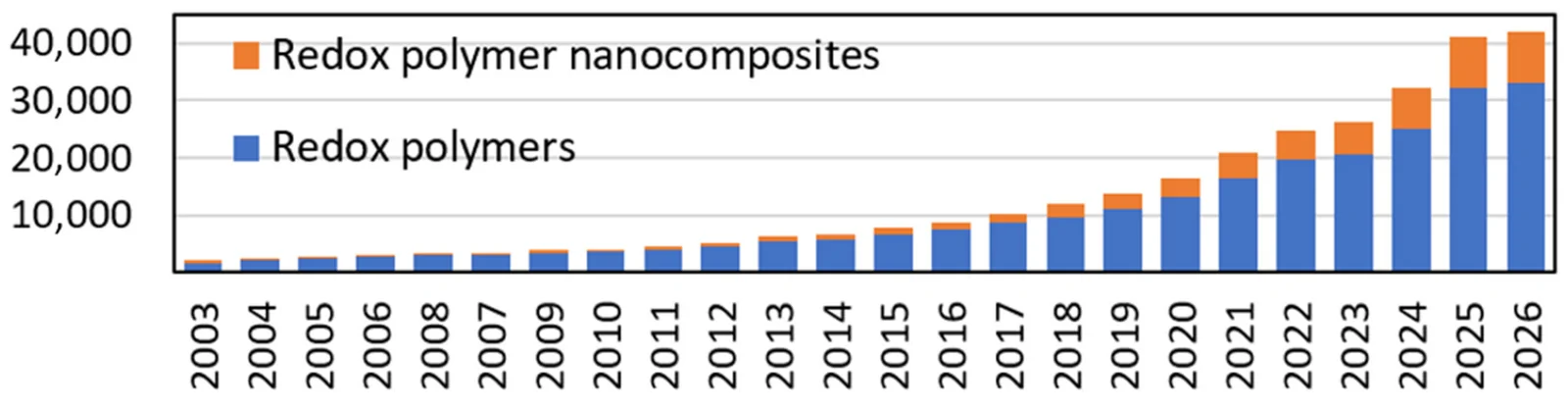
Number of publications in the ScienceDirect database per year with redox polymers and redox polymer nanocomposites as keywords.

The development of redox polymers offers a solution to this problem. These polymers function simultaneously as an immobilizing matrix, integrated mediator, and stabilizing medium for biological molecules [11,12,13,14]. Biomolecules are fixed inside the polymer chain, preventing their leaching, and redox centers provide fast and efficient electron transfer. Current research is focused on the development of hybrid nanostructured materials that combine redox polymers, carbon nanomaterials, and metal nanoparticles [15]. These materials exhibit enhanced performance characteristics and have the potential to be used in the creation of biosensors with advanced analytical capabilities. The relevance of the research topic is confirmed by the high number of publications over the past years (Figure 1). At the same time, it is worth noting that publications on the topic of nanocomposites account for more than 25%, which indicates the active introduction of nanomaterials into redox polymers.

The purpose of this review is to analyze the latest advances in the development of biosensors based on redox polymers. We discuss the main classes of redox polymers and their synthesis, methods for immobilizing biomolecules, and approaches to obtaining hybrid materials. Unlike previous reviews, this paper analyzes all three main types of redox centers—metal complexes, metallocenes, and organic radicals—in a single structure with an assessment of their effectiveness not only in terms of electron transfer kinetics but also taking into account biocompatibility, environmental impact, and scalability. In addition, we identify existing gaps that hinder the transition of these advanced materials from laboratory prototypes to commercial developments.

## 2. Structure of Redox Polymers

Much attention in the literature is paid to redox-active group-embedded polymers; in this review, we will focus on redox-active pendant-bearing polymers. Redox polymers represent complex hierarchical systems comprising four essential components:

- Polymer backbone—determines the mechanical properties and chain mobility (e.g., poly(vinylpyridine (PVP)) [16], polyethylenimine (PEI) [17], chitosan [18], bovine serum albumin (BSA) [19], and others);
- Redox-active centers—enable electron transport (e.g., [Os(bpy)_2_Cl]^2+^/^3+^ [20], ferrocene (Fc) [21], and phenazines [22]);
- Linkers (spacers)—optimal length ranges from 8 to 15 atoms (C_2_–C_4_ alkyl chains, polyethylene glycol (PEG) chains, and C_8_–C_16_) [20,23,24,25];
- Functional groups—regulate solvation and immobilization (-NH_2_, -COOH, -OH, and N-hydroxysuccinimide (NHS) esters) [11].

**Figure 2:**
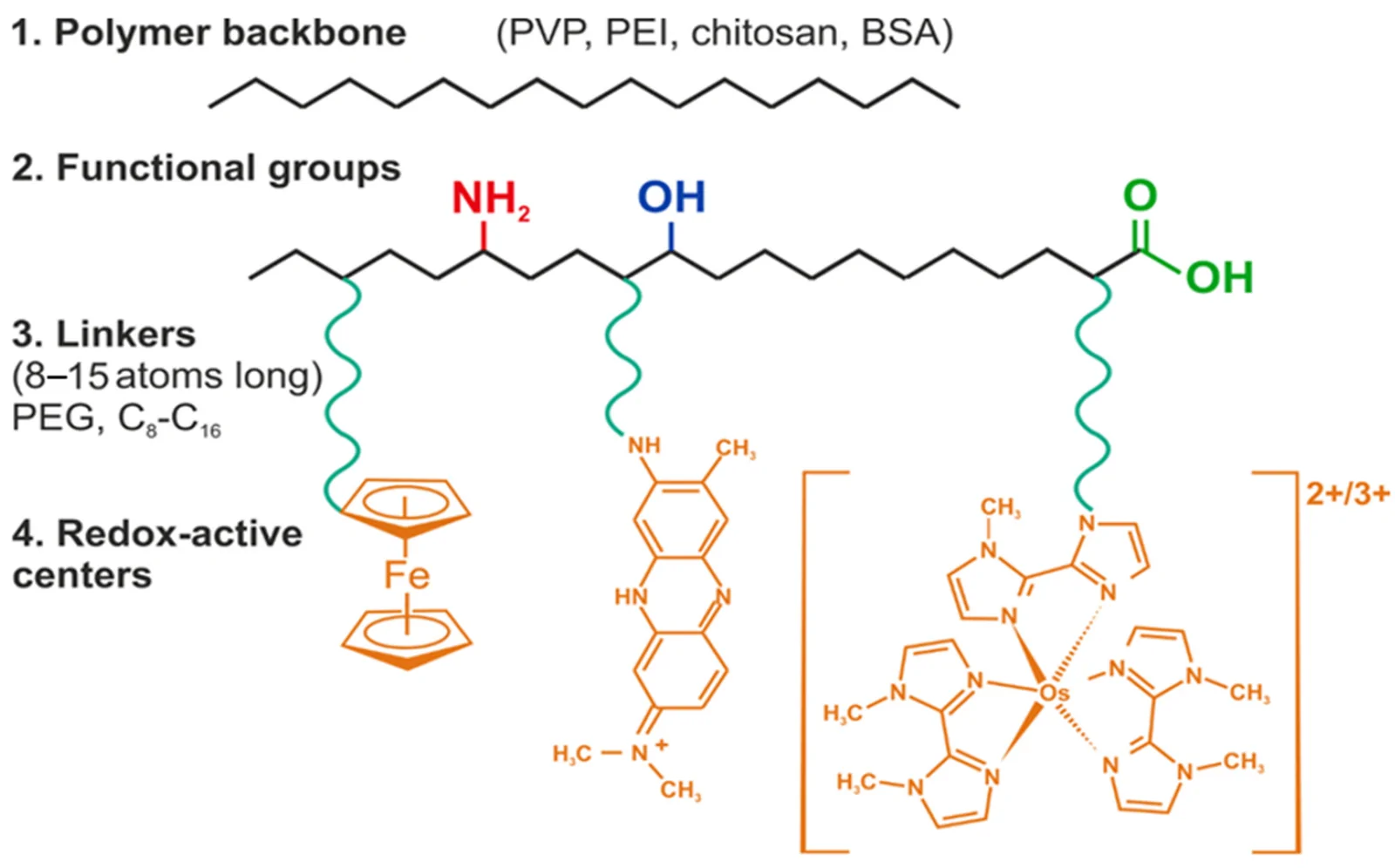
General architecture of redox polymers.

A schematic representation of redox polymer architecture is shown in Figure 2.

**Figure 3:**
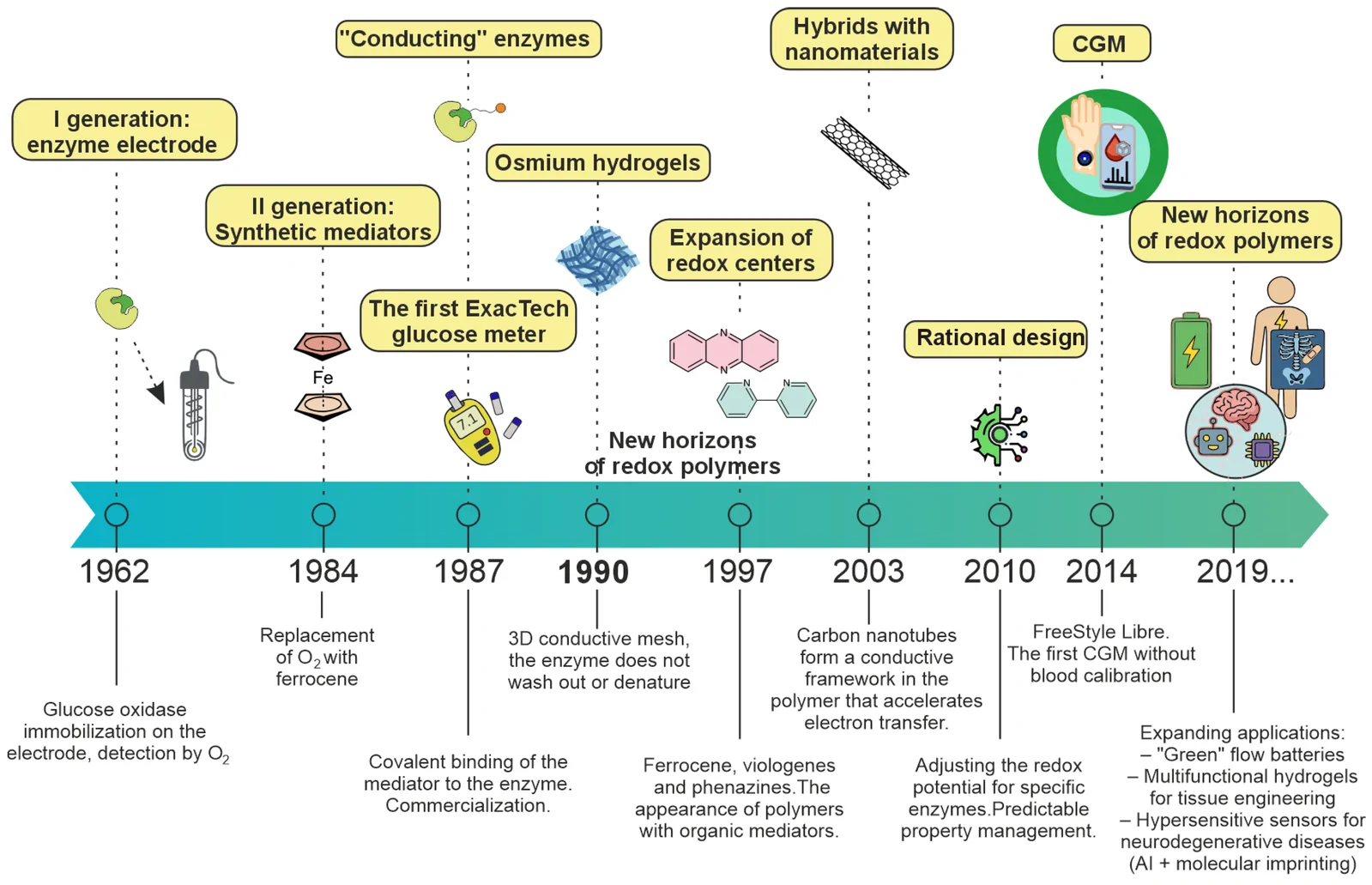
Timeline of redox polymers’ evolution in electrochemical biosensing.

The evolution of redox polymers is intrinsically linked to the development of the biosensor concept itself (Figure 3). The first biosensor, known as the “enzyme electrode,” was introduced by Clark and Lyons in 1962 [26]. Glucose oxidase (GOx)was immobilized on an electrode surface, and analyte detection was achieved by measuring changes in oxygen concentration (first-generation biosensors). This system was oxygen-dependent, prompting Cass and colleagues in 1984 to replace oxygen with synthetic mediators—Fc—giving rise to second-generation biosensors; however, mediator leaching from the electrode remained problematic [27].

In 1987, Adam Heller’s group solved this issue by covalently attaching the mediator to the enzyme, creating a “wired” enzyme [28]. That same year, Medisense Inc. launched the first glucose meter, ExacTech [29]. In the early 1990s, Heller developed osmium-based hydrogels, three-dimensional conductive networks that prevented both enzyme leaching and denaturation [30]. The research community soon expanded the range of redox centers. Beyond osmium, polymers modified with Fc [31] as well as organic mediators such as viologens [32] and phenazines [33] were actively developed. In the 2000s, carbon nanotubes (CNTs) were incorporated into polymers to create highly conductive pathways [34,35]. By the 2010s, rational design had emerged, enabling the tuning of redox potential for specific enzymes [36,37].

In 2014, Abbott (which had acquired TheraSense, also founded by Heller) launched the FreeStyle Libre system, the world’s first continuous glucose monitor requiring no blood calibration and utilizing “wired” enzymes based on redox hydrogels. The release of FreeStyle Libre marked the culmination of decades of research, confirming the enormous potential of redox polymers. Over the subsequent decade, these materials transformed from specialized glucose sensor components into a universal platform. Today, they underpin the development of “green” redox flow batteries [38], multifunctional hydrogels for tissue regeneration [39], and ultra-sensitive sensors for diagnosing neurodegenerative diseases (e.g., Alzheimer’s disease) employing artificial intelligence [40]. Thus, having progressed from simple enzyme electrodes to hybrid nanostructures, redox polymers continue to expand the frontiers of bioelectrochemistry and personalized medicine.

### Electron Transfer in Redox Polymers

Redox polymers belong to the class of polymers in which charge transport occurs predominantly via a “hopping” mechanism [41]. This type of electron transfer takes place between charge carriers localized at discrete molecular sites. Consequently, electronic conductivity in these materials arises from delocalized electronic states, with charge distributed across multiple monomer units of the polymer chain [42].

**Figure 4:**
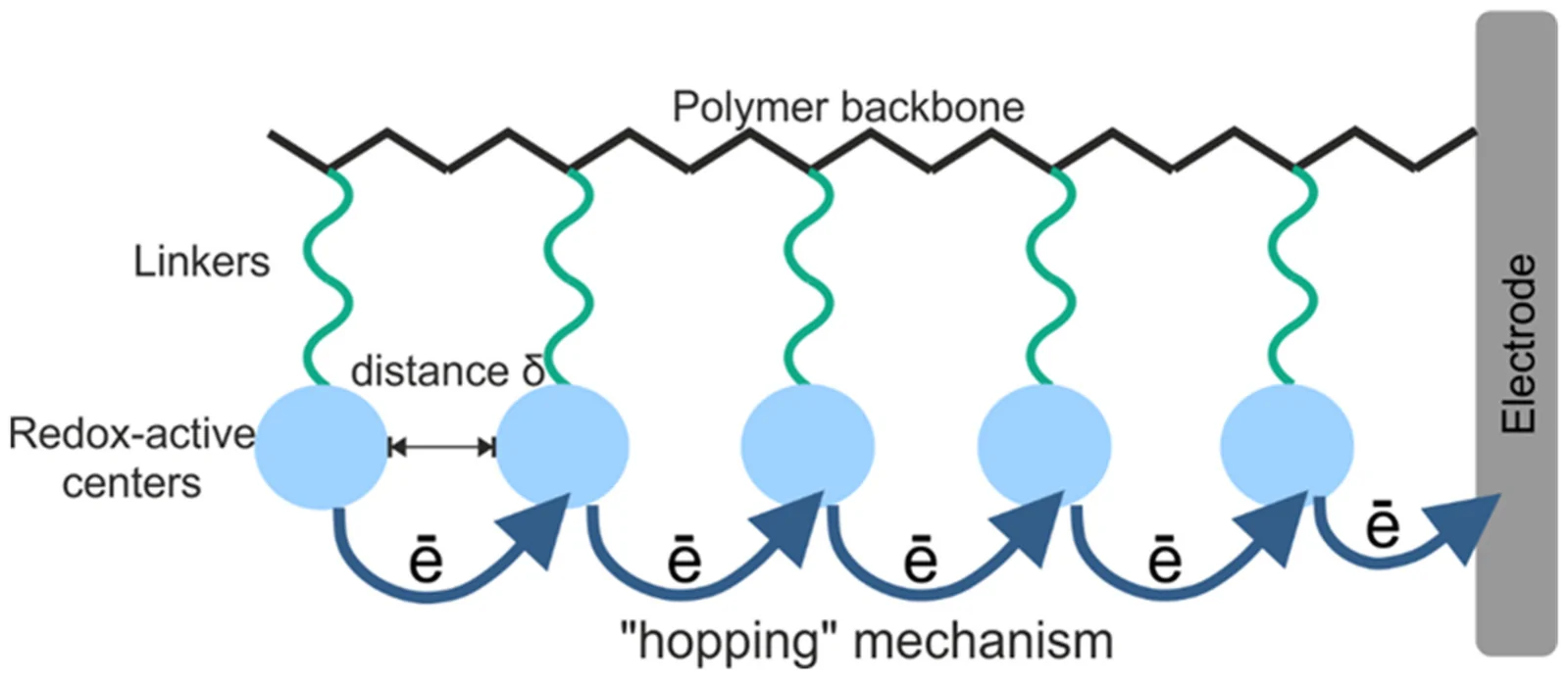
Schematic representation of electron transfer in a redox polymer.

In conducting polymers, charge carriers are generated through interactions involving double bonds and heteroatoms bearing lone electron pairs [43]. In contrast, for redox polymers, charge carriers are localized directly on the redox centers. Thus, spontaneous electron exchange occurs through interactions between oxidation–reduction centers attached to the polymer backbone [41,44]. This process is schematically depicted in Figure 4: the oxidized and reduced forms of the redox pair approach each other to a distance δ, defined by Marcus theory, at which electron transfer becomes feasible [11]; subsequent oxidation–reduction transformations enable electron transport along the polymer chain. Marcus theory is of particular importance for redox polymers, as it explains how reorganization energy and inter-center distance influence the electron diffusion coefficient (D_e_), enables the prediction of electron transfer efficiency from enzymes, and serves as a foundation for rational design, from selecting redox center potentials to calculating linker lengths and accounting for microenvironments within the polymer matrix.

Electron diffusion is maximal when the concentrations of reduced and oxidized fragments are equal, i.e., at the formal potential of the redox system [41,42]. It slows considerably when the vast majority of redox centers are either oxidized or reduced, meaning that electronic conductivity is low at potentials deviating from the redox potential of the system.

Electron transfer in hybrid “analyte–biomolecule–redox polymer–electrode” systems represents a multi-stage process comprising several interconnected steps. Upon immersion of the modified electrode into the analyte solution, target molecules diffuse from the bulk solution to the biosensor surface. Interaction with the biomolecule, such as an enzyme, triggers a specific biochemical reaction at the active site [45].

The reduced biomolecule subsequently transfers electrons to nearby redox centers within the polymer matrix, a critical step for enzymes with deeply buried active sites, such as GOx, for which direct electron transport to the electrode is hindered [11,46].

Within the polymer network, electrons migrate between redox-active groups via the hopping mechanism described above. The migration rate is governed by the distance between redox centers, polymer chain flexibility, the electron diffusion coefficient (D_e_), and the density of redox groups [11,47,48]. For example, D_e_ values in efficient systems based on osmium or ruthenium complexes with PVP reach 10^−8^–10^−7^ cm^2^/s, enabling rapid transport to the electrode surface [11].

Linker length exerts a complex and not always predictable influence on polymer transport properties. The seminal work by Mao et al. [20] increasing the amount of linker for osmium redox polymers to 13 atoms increases D_e_ by an order of magnitude due to increased mobility and accessibility of redox centers. Hossain et al. [23] confirmed that an “adequate spacer length” is necessary for rapid electron transfer. However, Easley et al. [25] showed that this dependence may be nonlinear and redox-pair-specific: for one redox pair, the transfer rate is nearly independent of linker length, while for another, transfer efficiency increases only up to a certain linker length, after which it declines. Polymer chain flexibility determines the segmental mobility and the ease with which redox centers approach each other to the distance required for electron “hopping.” Mao et al. [20] found experimentally that long flexible spacers provide excellent mobility of the centers, thereby accelerating movement. However, this advantage is not absolute and depends on the nature of the redox center, environmental conditions, and the degree of polymer crosslinking. Easley et al. [25] add an important nuance: the polypeptide chain itself establishes the overall electron transfer mechanism, while linker flexibility merely modulates the rate without altering its essence. Redox center density requires optimization. Excessively high density (short linkers) may restrict mobility and accessibility for the enzyme. Huang et al. [24] optimized linker length specifically to enable redox groups to “reach” the deeply buried active center of the enzyme, sacrificing density for accessibility.

This analysis demonstrates that optimizing charge transfer requires a delicate balance between linker length, chain flexibility, and center density. Contemporary research is addressing this challenge using machine learning methods. Alessandri et al. [49] employed multiscale modeling with machine learning surrogate models to predict the electron diffusion coefficient in phthalimide-containing polymers, reporting a three-order-of-magnitude increase compared to reference systems. An even more compelling approach was reported by Ganti et al. [50], where a machine learning platform not only predicts key characteristics of organic electrodes (voltage and specific capacitance) but also enables “inverse design” (selecting polymer structures for desired parameters by screening millions of potential candidates). These studies illustrate the transition from intuitive trial and error to rational design of materials with programmable properties.

The final stage of the process is heterogeneous electron transfer from the polymer to the electrode, generating a measurable current proportional to the analyte concentration [51,52,53]. Functionalization of electrode surfaces with CNTs and other nanomaterials significantly increases the effective conductive surface area, creating a developed architecture for redox polymer immobilization. This approach leads to increased redox center density and improved electron transport, as confirmed by studies demonstrating enhanced electrochemical responses in biosensor systems [15,41,53,54,55,56].

An analysis of the literature reveals a crucial trade-off in the design of redox polymers: optimizing for a high electron diffusion coefficient (D_e_) via long, flexible spacers often compromises the volumetric density of redox centers, thereby reducing the overall current density. Conversely, maximizing center density by using short linkers restricts chain mobility and can hinder efficient “hopping” due to steric constraints and increased reorganization energy, as described by Marcus theory [11]. Therefore, the reported D_e_ values (spanning from 10^−10^ to 10^−7^ cm^2^/s, Table 1) must be evaluated not in isolation but together with the redox center loading and the polymer’s swelling ratio in the working buffer. Notably, the application of machine learning models [49,50] marks a paradigm shift from empirical screening to rational design. However, a significant challenge remains: most computational models predict properties for ideal, defect-free polymers in silico, while experimental systems often suffer from non-uniform crosslinking and variable degrees of functionalization, leading to discrepancies between predicted and measured D_e_. Future modeling efforts must incorporate parameters for non-ideality and film morphology to become truly predictive for practical biosensor fabrication.

**Figure 5:**
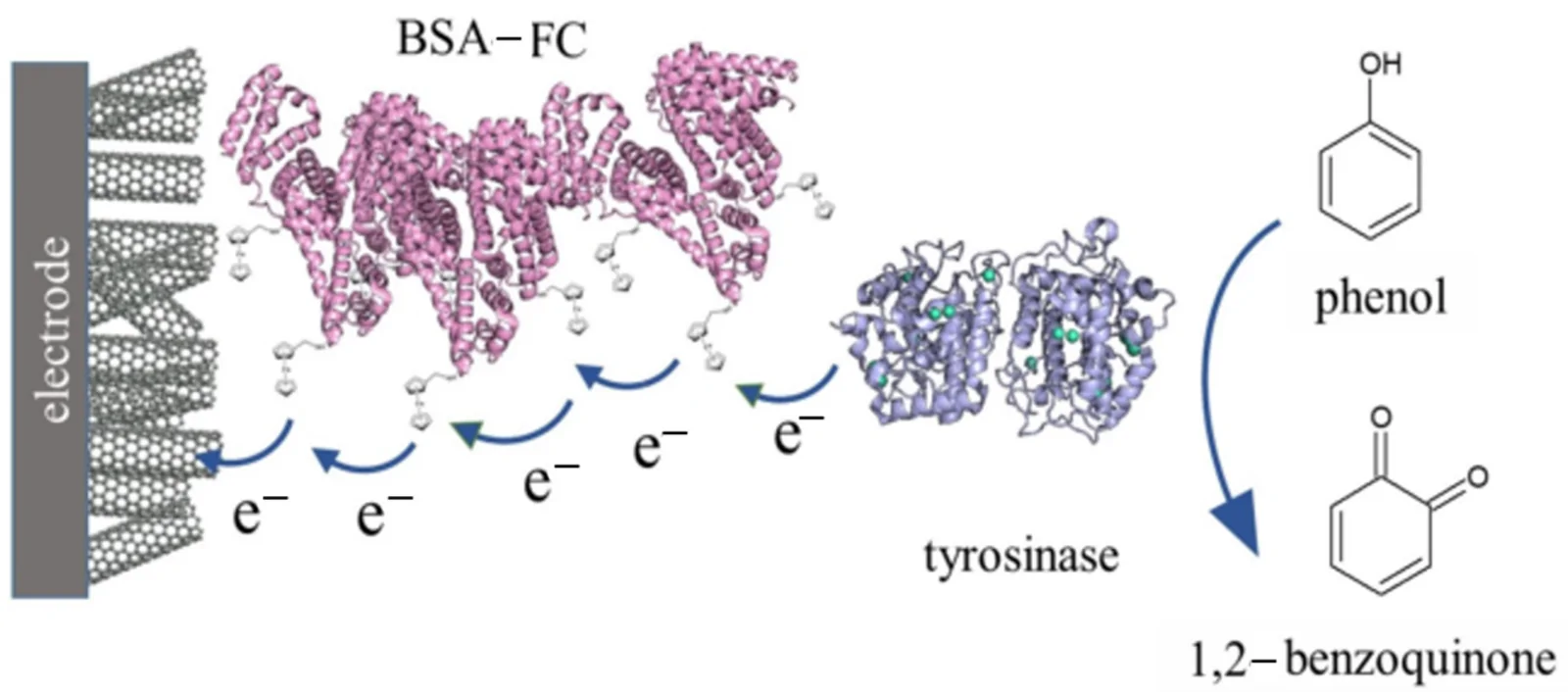
Model of electron transfer in the BSA-Fc-SWCNT-tyrosinase system. Reproduced with permission from ref. [56]. Copyright © 2024, the author(s), under an exclusive license to Springer Science Business Media, LLC, part of Springer Nature.

To illustrate the stages of electron transfer, Figure 5 presents a model for phenol detection using tyrosinase in conjunction with a redox polymer based on BSA modified with Fc as the redox center [56].

The efficiency of the entire cascade electron transfer pathway depends on the appropriate selection of the mediator’s redox potential, which must optimally match both the active site potential of the biomolecule and the operating potential of the electrode. The reversibility of redox reactions in polymers, controlled by an external potential, serves as a fundamental principle for numerous applied technologies. This principle underlies modern electrochromic displays, polymer-based batteries, and the construction of high-performance biosensors and bioelectrochemical energy converters [57].

## 3. Redox Centers

Redox polymers can be classified according to the type of redox-active centers they contain. Based on a systematic analysis of the literature, three major classes of redox centers are distinguished:

- Metal-containing complexes;
- Organometallic polymers;
- Polymers with organic redox groups (Table 1).

**Table 1:** Comparative characteristics of metal-containing, organometallic, and organic redox-active centers.

Redox Centers	Examples	D_e_, cm^2^/s	Potential, B	Key Advantages/Disadvantages	Sources
Metal-containing	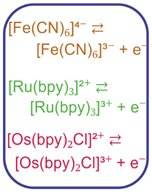	10^−10^–10^−7^	+0.05… +1.2	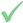 Fine-tuning the potential 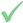 Fast kinetics 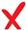 Cost, possible leaching 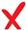 Sensitivity of the coordination sphere to pH and competitive ligands	[11,13,58,59,60,61,62,63,64,65,66]
Organome-tallic	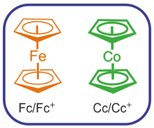	10^−9^–10^−7^	−1.1… +0.9	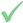 Fast transfer 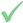 Biocompatibility 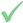 Stable M-C connection 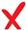 Polymer mass	[21,53,57,66,67,68,69,70,71,72,73]
Organic	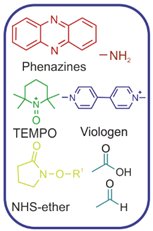	10^−10^–10^−5^	−0.8… +0.8	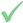 Ease of synthesis 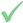 Structural diversity 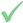 Biocompatibility 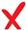 Low conductivity 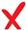 Tendency to dissolve	[57,61,74,75,76]

The comparative data in Table 1 highlight that no single class of redox centers universally outperforms the others. The choice is application-dependent. Metal-containing complexes (e.g., Os and Ru) are the materials of choice for fundamental studies and high-performance biofuel cells due to their unmatched stability and fast kinetics. However, their high cost and potential toxicity significantly limit their use in implantable and disposable diagnostics. Organometallic centers (e.g., ferrocene) strike the best balance for the majority of oxidase-based clinical biosensors. They offer a wide potential window, good biocompatibility, and relatively low cost, making them ideal candidates for commercial point-of-care devices. The key challenge remains optimizing their attachment to prevent gradual leaching during prolonged operation.

Organic radicals (e.g., TEMPO and phenazines) represent the most promising direction for future “green” and implantable biosensors. Their ease of synthesis, structural diversity, and biocompatibility are unparalleled. However, to achieve their full potential, significant effort is still required to overcome their lower intrinsic conductivity and the tendency for radical centers to degrade through side reactions during potential cycling. This classification underscores that the selection of a redox center must be a deliberate, problem-specific decision, integrating electrochemical performance with economic and biological safety requirements.

### 3.1. Metal-Containing Complexes

**Figure 6:**
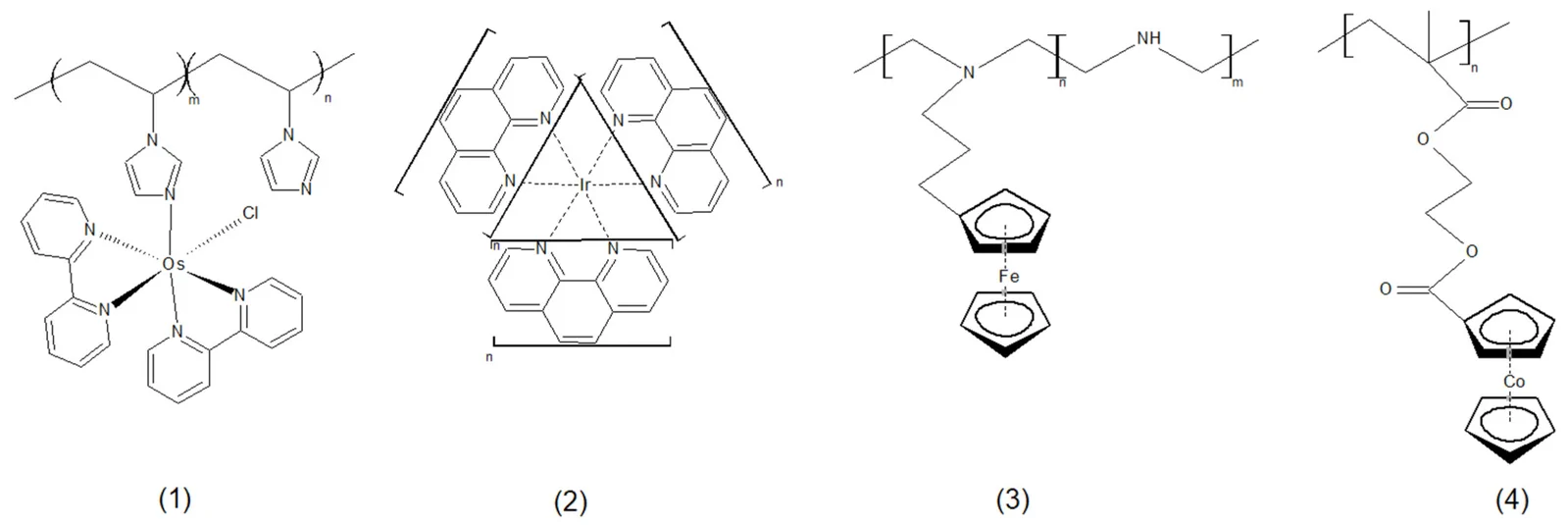
Examples of polymers containing metal-containing complexes and organometallic redox centers: osmium complexes (**1**), iridium complexes (**2**), Fc (**3**), and cobaltocene (**4**).

Among metal-containing redox polymers, osmium complexes (Os^2+^/^3+^) are most frequently employed (Figure 6). The literature predominantly describes osmium complexes with pyridine (py), 2,2′-bipyridine (bpy), and their derivatives, as well as with di-N-alkylated-2,2′-diimidazole [11,58,59,60], with the [Os(bpy)_2_Cl]^2+^/^3+^ system being the most thoroughly characterized [11,61].

The incorporation of Os, Ru, Fe, Zn, Ir, Cu, and Pt ions into polymer matrices imparts unique functional properties, including catalytic activity, reversible redox transformations, magnetism, and luminescence [62]. For instance, ruthenium complexes with bpy—[Ru(bpy)_3_]^2+^—exhibit luminescent properties and find application in optical sensors [11,63]. Ruthenium complexes are also known to bind nucleic acid molecules [64,65]. Iron-based complexes include hexacyanoferrates (Prussian blue) and cytochrome centers [66].

Zinc(II)-based polymers represent another well-studied example, owing to the economic accessibility of zinc. Meanwhile, iridium(III) complexes within polymer matrices are of particular interest for the development of efficient optoelectronic devices (Figure 5) [62].

For successful biosensor applications, the key criteria for selecting a metal-containing complex are biocompatibility and absence of toxicity, as the material is in direct contact with the biological environment.

### 3.2. Organometallic Redox Centers

The most stable and thoroughly studied system in the organometallic redox polymer class is Fc/ferrocenium (Fc/Fc^+^). Fc is widely employed in redox polymers due to its unique combination of electrochemical and physicochemical properties: a tunable redox potential that allows system adjustment for specific enzymes or analytes; stability over a broad pH range; and low toxicity and biocompatibility compared to heavy metal complexes (e.g., osmium) [67,68,69,70,71]. The best-characterized Fc-based polymers include poly(vinylferrocene) (PVF) [69,70], composites with polythiophene and ferrocene–polypyrrole [69,72], derivatives with chitosan [69], polyethylenimine-based derivatives (Figure 5, [21,73]), and dendrimer-containing systems [69]. Fc-based redox polymers are primarily employed as mediators for oxidases, with optimal performance observed in combination with nanotube-based composite materials [56].

Cobaltocene (Cc/Cc^+^) [57,62] and metalloporphyrins—heme-like structures for biomimetic applications [53]—are less common as organometallic redox-active polymers (Figure 5).

### 3.3. Organic Redox Centers

**Figure 7:**
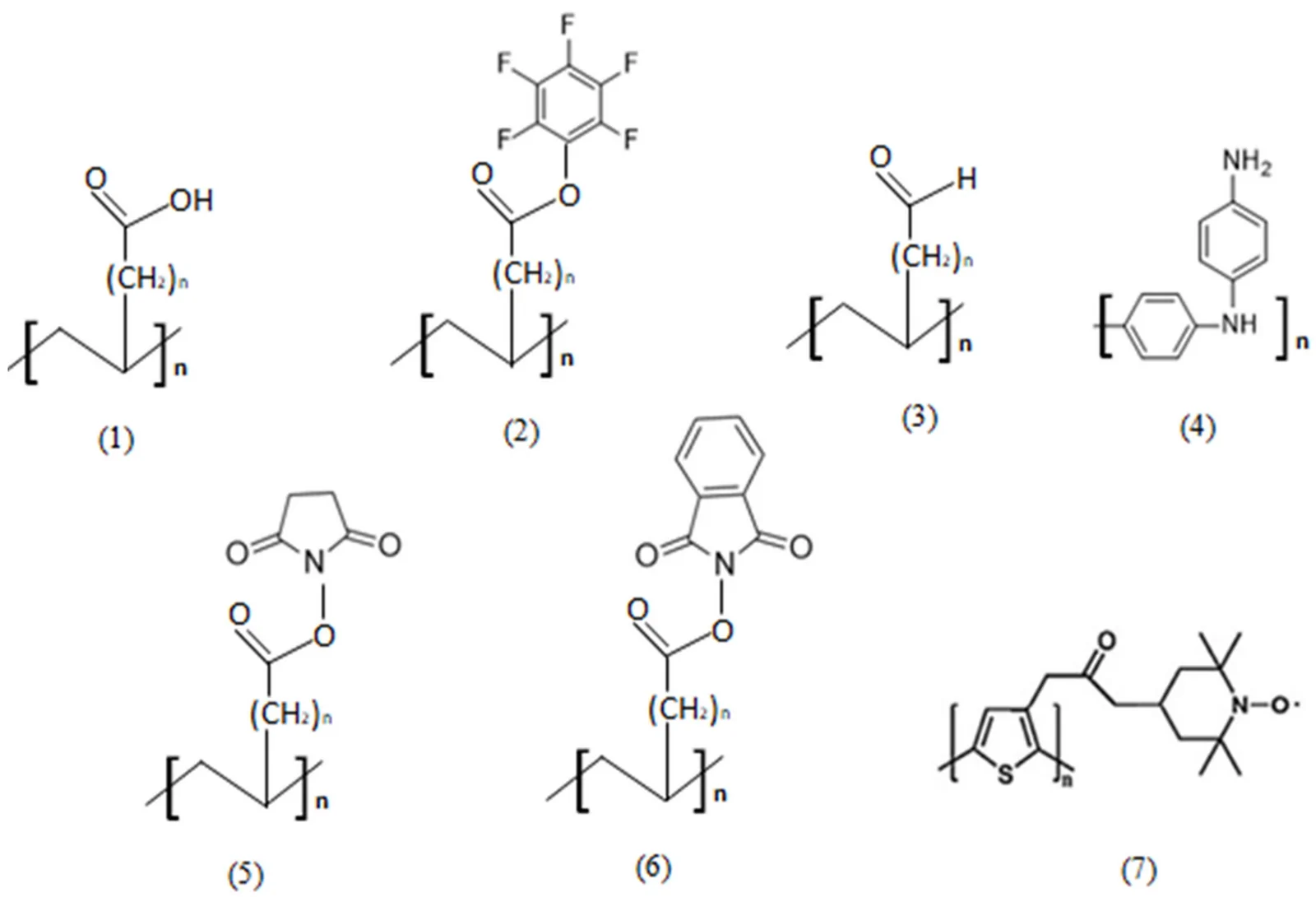
Examples of organic redox centers: (**1**) carboxyl groups, (**2**) pentafluorophenyl esters, (**3**) aldehyde groups, (**4**) amino groups (including aromatic amines), (**5**) NHS esters, (**6**) N-hydroxyphthalimide esters, and (**7**) TEMPO.

In biosensors, organic redox centers include compounds bearing amino, carboxyl, and aldehyde groups; carboxylic acid esters with NHS, N-hydroxyphthalimide, or pentafluorophenyl groups, which are commonly used to functionalize polyamines, polypyrroles, and polythiophenes (Figure 7) [61]. Quinones—primarily anthraquinone, naphthoquinone, and benzoquinone—are also employed [57,61].

Nitroxyl radicals, particularly 2,2,6,6-tetramethylpiperidine-1-oxyl (TEMPO) (Figure 7), constitute a promising class of organic redox centers. TEMPO-based polymers are characterized by rapid charge transfer [75,76], leading to their application in organic battery cathodes, supercapacitors, and biosensors [57]. To fine-tune functionality, block copolymers and hybrid systems have been developed in which TEMPO groups are incorporated into conjugated chains (e.g., polythiophene and poly(3,4-ethylenedioxythiophene) (PEDOT)), resulting in synergistic integration of polymer chain conductivity and radical redox activity [76]. TEMPO-containing polymers have also been applied in disease diagnostics: incorporation of TEMPO into a polymeric deoxyribonucleic acid matrix creates a magnetic resonance imaging contrast sensor for early detection of osteoarthritis [77].

**Figure 8:**
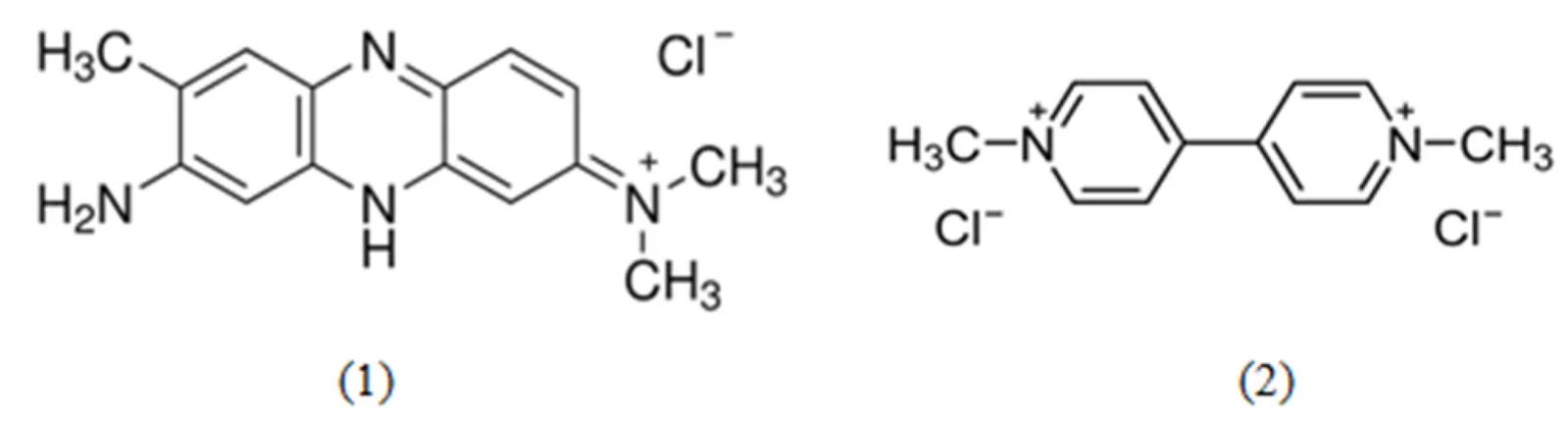
Representative phenazines and viologens: (**1**) neutral red and (**2**) methyl viologen (paraquat).

Phenazines (neutral red and Janus green), phenothiazines (methylene blue and thionine), and viologens—biologically relevant structures capable of multi-electron transfer—have also found applications in biosensors (Figure 8) [57,61,74]. Phenazines are compatible with NADH-dependent dehydrogenases and are used in biosensors for glucose, lactate, and ethanol detection [61].

Although osmium complexes do not hold the absolute record for electron diffusion coefficient values (being outperformed by some organic systems), they remain unrivaled in terms of stability, reversibility, and efficiency of enzyme “wiring” under physiological conditions. However, their potential toxicity and high cost limit their application in implantable and disposable devices, where biocompatibility requirements are paramount. In this context, Fc and, particularly, organic radicals (TEMPO) are becoming the preferred choice, offering an optimal balance between biocompatibility, cost, and electrochemical performance, despite somewhat lower electron transfer rates in certain systems.

## 4. Polymer Matrices

**Figure 9:**
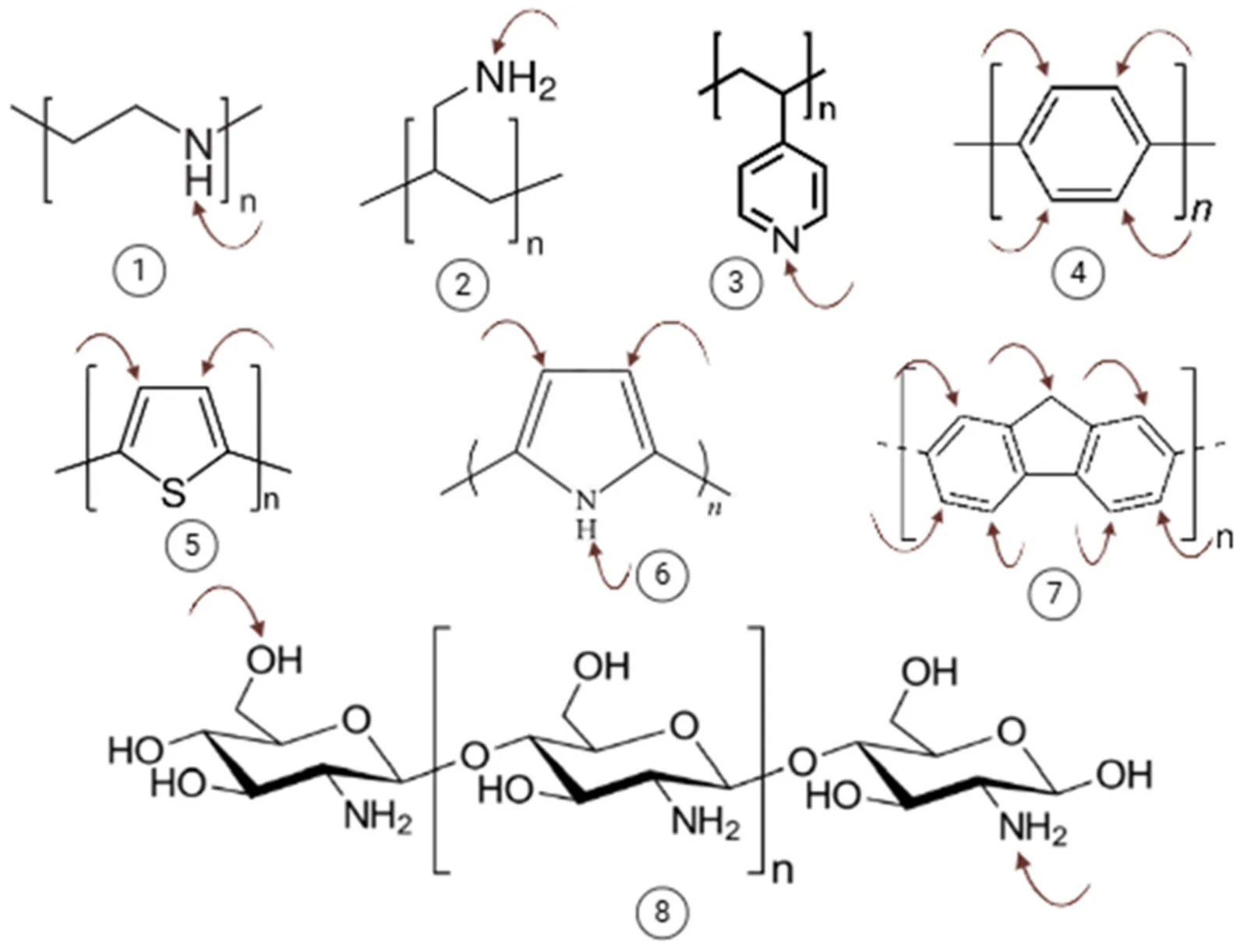
Examples of polymer matrices and their primary interaction sites: (**1**) poly-ethy-lenimine, (**2**) poly(allylamine), (**3**) poly(vinylimidazole), (**4**) poly(p-phenylene), (**5**) polythiophene, (**6**) polypyrrole, (**7**) polyfluorene, and (**8**) chitosan.

Inorganic polymers suffer from several disadvantages, including protracted synthesis, difficulty in optimizing the stoichiometric ratio of starting reagents, and mechanical fragility. Consequently, high-molecular-weight organic polymers are more convenient (Figure 9), as their crosslinking with mediators is straightforward and rapid [19].

The selection criteria for the polymer backbone include the following:

- Molecular weight: 10–100 kDa;
- Chain flexibility (influences segmental dynamics and electron diffusion coefficient D_e_) [11];
- Functional groups (e.g., -NH_2_, -COOH, and -OH) for covalent attachment of redox centers or linkers and for biomolecule immobilization;
- Solubility (determines the electrode deposition method);
- Biocompatibility (for interaction with biological molecules) [11,12].

### 4.1. Vinyl Polymers

Among polymer backbones for redox polymers, vinyl polymers are the most widely used. PVP serves as a platform for osmium and ruthenium complexes due to the ability of pyridine rings to coordinate metal ions [53,58]. Poly(vinylimidazole) is also employed to create pH-sensitive systems.

### 4.2. Flexible Linear Polymers

For the construction of flexible structures, linear polymers such as PEI or poly(allylamine) are used, which contain primary and secondary amino groups for covalent immobilization [21,73].

### 4.3. Conducting Redox Polymers

**Figure 10:**
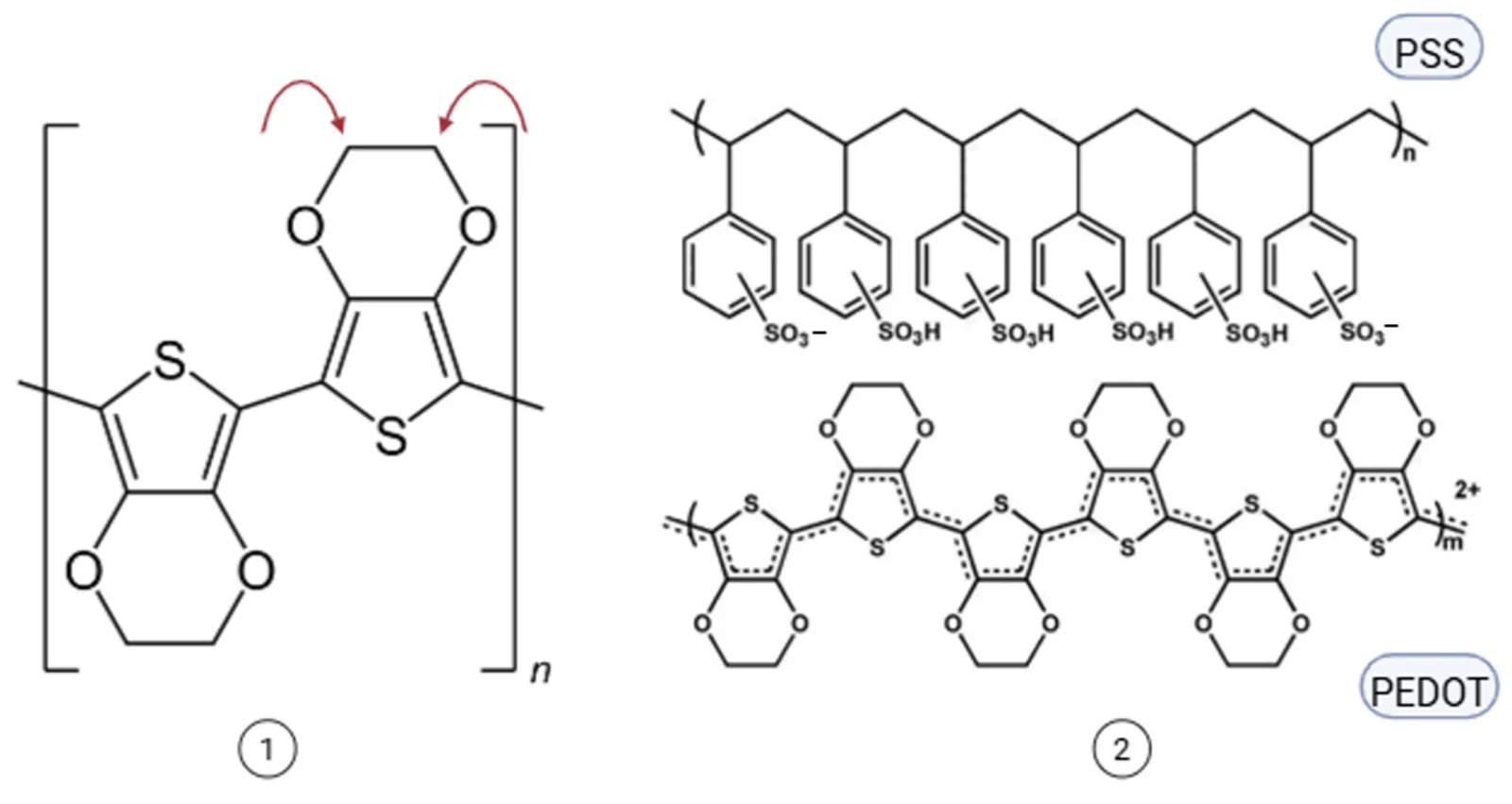
Strategies for PEDOT modification: (**1**) modification of the ethylenedioxy bridge; (**2**) introduction of Poly(styrenesulfonate).

Of particular interest are conducting redox polymers, which consist of a conducting polymer backbone bearing appended redox-active side groups. Beyond those already described, conjugated polymers find application, including polyfluorenes (possessing photo- and electroluminescent properties), polythiophenes (whose structure is readily modified and allows efficient binding to biological macromolecules), and poly(p-phenylene)s (with rigid backbones for constructing ordered systems) [57,61]. Polypyrrole (PPy), characterized by high electrical conductivity and amenability to electrochemical deposition, is also widely used [57,78], as is PEDOT, which exhibits exceptional stability (Figure 10) [79,80]. Furthermore, Karlsson et al. [81] demonstrated that incorporating redox-active groups into the structure of the conducting polymer PPy can not only impart new properties but also alter the backbone properties through electron transfer between redox groups and the main chain. Such interactions can either enhance or degrade system performance; therefore, elucidating the design principles of conducting redox-active polymers is of great value.

### 4.4. Natural Polymers

Natural polymers are particularly valuable resources for fabricating redox polymers used in biomedicine, including implantable biosensors and targeted drug delivery systems, owing to their biocompatibility [82]. Both polysaccharides and protein molecules have been reported in the literature.

Dextrans, which are branched polysaccharides, possess a high density of hydroxyl groups. This enables diverse chemical modifications, including the introduction of redox-active centers (osmium complexes [11], Fc [70], or organic redox groups [57]), as well as the formation of hydrogels with a high degree of swelling in aqueous media, thereby facilitating substrate and product diffusion to and from immobilized biomolecules [83].

Chitosan, a biocompatible and biodegradable natural polymer, represents a promising platform for functional coatings [84,85]. Yan et al. [85] developed an electrochemical biosensor based on alkaline phosphatase in which signal amplification is achieved using a redox condenser based on a catechol–chitosan polymer coating on the electrode.

Protein molecules, such as BSA, are also employed as natural polymer matrices. The use of BSA is justified by its biocompatibility with various enzymes and the presence of primary amino groups for attaching redox centers and other modifications [19,56].

### 4.5. Dendrimers

Contemporary research also employs dendrimers—highly ordered polymeric macromolecules with symmetrically branched structures surrounding a multifunctional central core—such as poly(amidoamine) (PAMAM) [86].

The characteristics of polymer matrices and their interaction sites are summarized in Table 2.

### 4.6. Summary and Comparative Analysis

Vinyl polymers (PVP and poly(vinylimidazole)) represent the classic platform for metal-complex redox systems due to their ability to coordinate metal ions, but their application is limited by non-specific adsorption. Conducting polymers, particularly PEDOT and PPy, combine high conductivity and stability; however, the unpredictability of their properties during modification requires optimization, since it depends on interdependent parameters—the level of doping, phase morphology, and solvation—which must be systematically controlled for rational material design. [81]. Flexible linear polymers (PEI and Poly(allylamine)) and natural matrices (chitosan, dextran, and BSA) are preferred for biomedical applications: flexible polymers provide high center density, while natural polymers offer biocompatibility and hydrogel formation, albeit with lower mechanical strength. Dendrimers with controllable group density remain primarily research materials due to their complex synthesis [86]. Thus, matrix selection is task-dependent: for maximum conductivity, vinyl and conducting polymers; for a balance of properties, flexible linear polymers; and for implantable devices, natural polymers, despite their limited stability.

In summary, the selection of a polymer matrix should follow a “performance vs. application” hierarchy:

- For maximum conductivity and facile electron transfer in biosensor prototypes, vinyl polymers (PVP and poly(vinylimidazole)) and conducting polymers (PEDOT) are the most effective, despite their synthetic complexity and potential for non-specific adsorption.
- For applications requiring high loading of redox centers and simple synthesis (e.g., basic research and early-stage sensor design), flexible linear polymers (PEI and poly(allylamine)) are optimal.
- For in vivo or implantable devices, natural polymers (chitosan, dextran, and BSA) are essential due to their biocompatibility and biodegradability. However, their use necessitates additional strategies to compensate for lower mechanical stability and limited chemical resistance, such as crosslinking with PEG-based agents or incorporation into nanocomposite films.

To select a polymer matrix for implantable devices, it is necessary to take into account not only the conductivity and biocompatibility but also the rate of degradation under physiological conditions (pH 7.4, 37 °C, enzymes, and reactive oxygen species), which for most polymers is determined by hydrolysis, radical oxidation, or enzymatic cleavage. For vinyl polymers (PVP and polyvinylimidazole), degradation proceeds mainly by a radical mechanism through the oxidation of heterocycles and cleavage of C–N bonds in quaternary ammonium groups, whereas conductive polymers (PEDOT and PPy) are susceptible to nucleophilic attack at β-positions in the oxidized state, which is accelerated by chloride ions of physiological solutions; PEDOT is especially vulnerable: PSS due to acid-catalyzed hydrolysis of PSS sulfogroups. Flexible linear polymers (PEI and polyallylamine) They are oxidized to imines, followed by chain cleavage, and natural polymers (chitosan, dextran, and BSA) are rapidly destroyed enzymatically (by lysozyme, dextranase, and proteases), which limits their service life. An informed choice of material requires in vitro testing (PBS + H_2_O/lysozyme, 37 °C), quantitative analysis of degradation products, and the development of protective coatings, since even the best conductive material without proven long-term stability is unsuitable for long-term implantations.

## 5. Synthesis of Redox Polymers

### 5.1. Chemical Synthesis

Chemical synthesis encompasses two main approaches: polymerization of monomers pre-functionalized with redox groups and post-functionalization of pre-synthesized polymers.

#### 5.1.1. Key Reagents

The choice of reagents is determined by the reaction medium and the intended application. The 1-Ethyl-3-(3-dimethylaminopropyl)carbodiimide (EDC)/NHS pair (EDC for carboxyl group activation and NHS for stabilization of activated esters) represents the “gold standard” for carboxyl group activation in aqueous media [1,21]. N,N′-Dicyclohexylcarbodiimide is used exclusively in anhydrous organic solvents for monomer and peptide synthesis [2]. Among initiators, azobisisobutyronitrile dominates in organic polymer backbone synthesis [20,87,88], while K_2_S_2_O_8_ is employed in aqueous systems, enabling hydrogel polymerization under mild conditions while preserving biomolecule activity [41,89]. Solvent selection is task-dependent: dimethylformamide is universal for synthesis and film deposition [5]; DMSO is preferred for biomolecule handling due to its biocompatibility; and acetonitrile remains the standard for electrochemical measurements [67]. For crosslinking, glutaraldehyde is a fast and reliable reagent [6,19]; however, for implantable devices, PEG-diglycidyl ether is often chosen, as it provides more flexible and biocompatible crosslinking that better preserves enzyme activity [11].

#### 5.1.2. Radical Polymerization

Polymer backbone synthesis is predominantly carried out via radical polymerization. Using azobisisobutyronitrile as an initiator, the synthesis of PVP proceeds as follows: The initiator decomposes into primary radicals at 60–70 °C [87,88]:

(CH_3_)_2_C(CN)-N=N-C(CN)(CH_3_)_2_ → 2 (CH_3_)_2_C(CN)• + N_2_ (60–70 °C) (1)

The resulting primary radicals attack the double bonds of vinyl monomers (chain initiation):

(CH_3_)_2_C(CN)• + CH_2_=CH-C_5_H_4_N → (CH_3_)_2_C(CN)-CH_2_-CH•(C_5_H_4_N) (2)

Subsequent sequential addition of monomer molecules to the active centers constitutes chain propagation. Denoting (CH_3_)_2_C(CN)-CH_2_-CH•(C_5_H_4_N) as P_1_•:

P_1_• + CH_2_=CH-C_5_H_4_N → P_1_-(CH_2_-CH-C_5_H_4_N)• (3)

Chain termination occurs predominantly via recombination of two macroradicals:

P• + •P → P−P(4)

where P• = (CH_3_)_2_C(CN)-[CH_2_-CH(C_5_H_4_N)]_n_-CH_2_-CH•(C_5_H_4_N).

Molecular weight is controlled by the [M]/[I] ratio, with a dodecylmercaptan chain regulator [90]. Copolymerization is also employed, for example, combining vinylpyridine with vinylpyrrolidone (70:30) [61]. Synthesized polymers are characterized by cyclic voltammetry (redox potential and center density), IR spectroscopy (amide bond formation), and NMR (degree of functionalization) [57,62,91,92,93,94].

#### 5.1.3. Post-Functionalization Strategies

The method of modifying monomers or pre-synthesized polymer matrices with redox-active groups depends on the nature of the starting materials. Post-functionalization typically requires reactive groups within the polymer chain. PEI-based systems with free amino groups are particularly effective for introducing organometallic and organic redox groups [21,72,73,93]. Free -NH_2_ groups can undergo condensation to form Schiff bases, a strategy frequently used for natural polymers such as chitosan and BSA [56].

For transition metal complexes (e.g., osmium compounds), incorporation into the polymer requires donor moieties within the chain. For instance, pyridine fragments of poly(N-vinylimidazole) displace chloride ions from the inner coordination sphere of osmium complexes [11].

Modification of heterocyclic monomers or polymers can also be achieved via N-alkylation, for example, using haloalkylferrocene derivatives. In the absence of heterocyclic atoms, polymers containing aromatic rings may be modified via electrophilic substitution, such as at the 3-position of pyrrole [95].

For the preparation of functionalized vinyl polymers, dehydration of hydroxyl derivatives of redox agents is employed. For example, poly(vinylferrocene) is obtained by synthesizing the vinylferrocene monomer followed by radical polymerization [96].

#### 5.1.4. Crosslinking and Linker Design

A key objective in electrochemical biosensor fabrication is stabilization of the active layer on the electrode surface. Crosslinking agents form robust three-dimensional networks within the polymer matrix, ensuring mechanical stability, preventing leaching, and securely retaining biologically active components, thereby significantly enhancing biosensor longevity and operational reliability. In cases where crosslinking agents are toxic, physical crosslinking may be employed [97].

The introduction of linkers for efficient electron transfer is of particular interest. Short, rigid linkers (e.g., C_2_–C_4_ alkyl chains) maximize redox center density and enable efficient hopping-based electron transfer. Long, flexible linkers (PEG chains and C_8_–C_16_) increase redox group mobility but reduce overall active center density [20,23,24]. Appropriate linker selection allows for fine-tuning of the electron diffusion coefficient, redox potential, and overall biosensor or catalytic system efficiency.

#### 5.1.5. Enzyme-Catalyzed Synthesis

The catalytic action of enzymes represents a mild and environmentally friendly alternative to traditional chemical methods. Redox enzymes such as peroxidase and laccase have been successfully employed to produce conducting polymers, particularly polyaniline [89]. An important feature of this approach is its flexibility: process parameters can be deliberately varied to control the properties of the resulting polymer [98].

### 5.2. Electropolymerization

**Figure 11:**
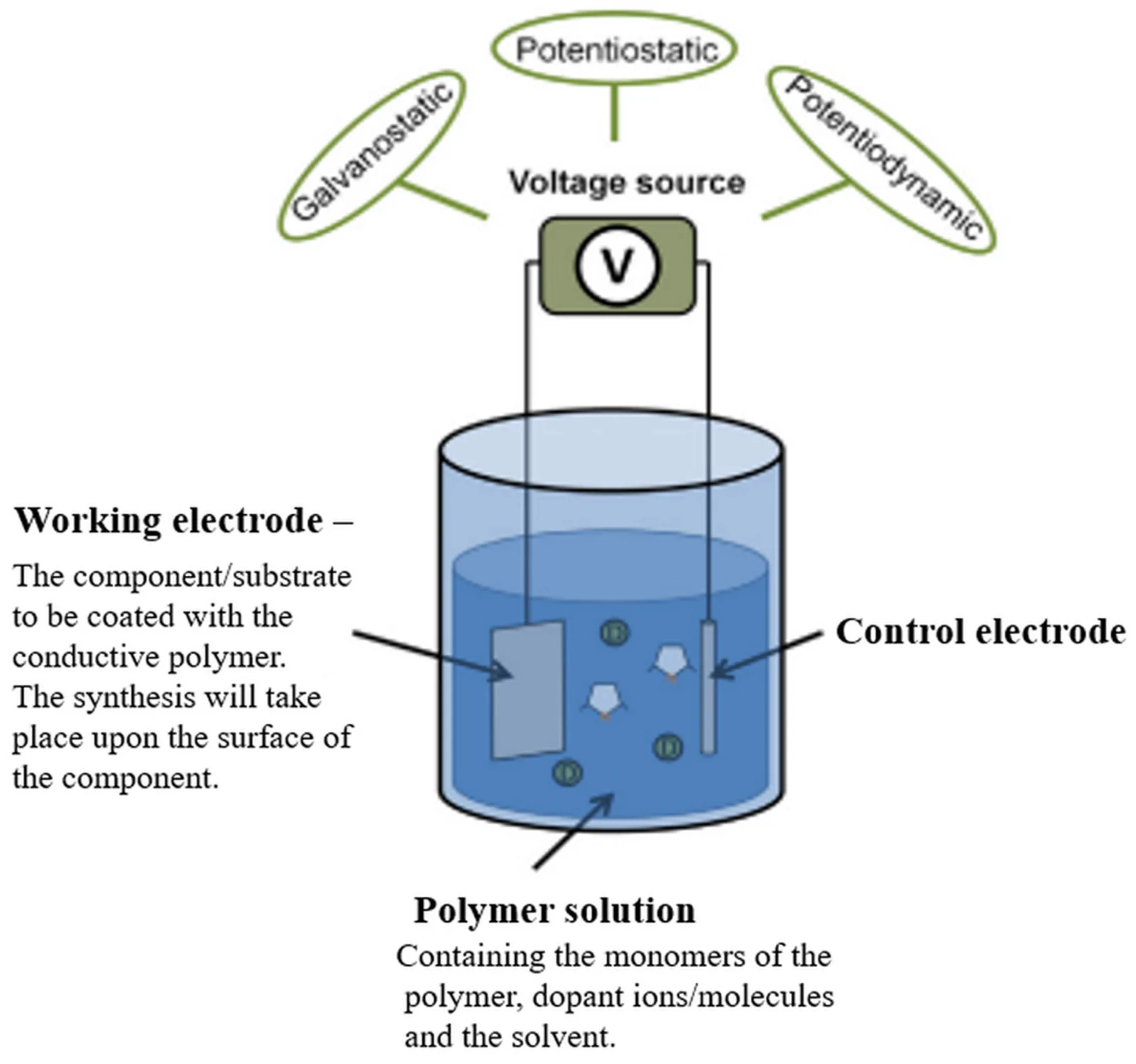
Schematic diagram of the electrochemical cell for redox polymer synthesis via electropolymerization. Reproduced with permission from ref. [99]. Copyright © 2019 by the authors.

Electropolymerization is a method for synthesizing polymer films directly on the electrode surface through electrochemical activation of monomers (Figure 11) [99]. The solution typically contains monomers, a solvent, and an electrolyte/salt [41]. The electrode potential is varied depending on the nature of the doping anion/cation and the polymerization medium.

Commonly used working electrode materials include chromium, gold, nickel, palladium, titanium, platinum, and indium tin oxide-coated glass slides. Semiconductor materials such as n-type silicon, gallium arsenide, cadmium sulfide, and semimetallic graphite are also employed for polymer film growth [99].

The process begins with oxidation or reduction of monomer molecules at the electrode surface, generating active radicals. Chain propagation occurs through sequential monomer addition to active centers, with growing chains remaining immobilized on the electrode surface. Chain termination can proceed via reaction with nucleophiles in solution or surface passivation upon reaching a critical film thickness [99,100].

Several electrochemical methods are employed in practice: galvanostatic (constant current density), potentiostatic (constant potential), and cyclic voltammetry [62,99,100]. Cyclic voltammetry enables precise process control by monitoring the increase in polymer film oxidation/reduction currents with each cycle. Film thickness is controlled by the number of cycles or the amount of charge passed. Film morphology strongly depends on the nature of the solvent, supporting electrolyte, and deposition potential. It is also difficult to scale, and the application of high potentials may lead to irreversible monomer oxidation [101].

#### 5.2.1. PEDOT Electropolymerization

**Figure 12:**
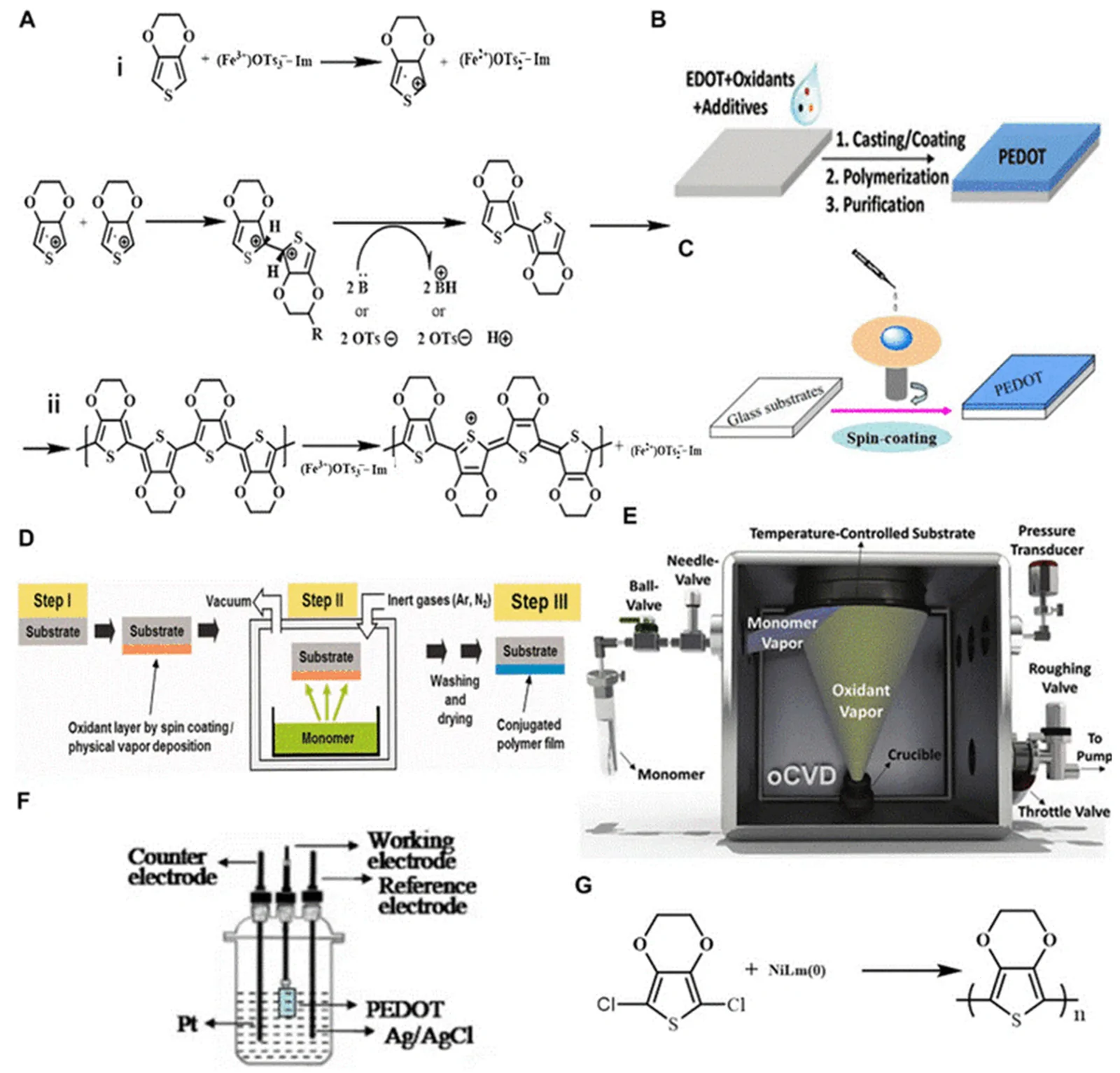
PEDOT synthesis: (**A**) oxidative polymerization; (**B**,**C**) in-situ polymerization, (**D**) flow chart of vapor-phase polymerization; (**E**) schematic diagram of reactor; (**F**) a three-electrode device for electrochemical polymerization; (**G**) coupling polymerization of transition metals. Reproduced with permission from ref. [102]. Copyright © 2021 Nie, Li, Yao, and Jin.

For example, PEDOT electropolymerization is conducted in a three-electrode cell, where oxidation of the EDOT monomer occurs at the working electrode under an applied potential (Figure 12) [102]. Lithium salts or ionic liquids are used as electrolytes, with their anions (ClO_4_^−^, PF_6_^−^, and TFSI^−^) incorporating into the polymer matrix as counterions, determining conductivity and morphology. By varying the electrolyte and deposition potential, films with conductivities up to 2000 S/cm and tailored optoelectronic properties can be obtained.

#### 5.2.2. Redox Polymer Films

In the context of redox polymer production, this method enables the fabrication of functionalized films through polymerization of a monomer bearing a pre-incorporated redox group (e.g., vinylferrocene electropolymerization) or through copolymerization of the main monomer with derivatives bearing redox-active centers and biological ligands. Copolymerization of pyrrole with pyrrole–ferrocene yields films with redox center densities up to 1.2 × 10^−8^ mol/cm^2^, which efficiently mediate electron transfer to oxidases [53].

#### 5.2.3. Biocompatible Surfaces

Electropolymerization is also employed to create biocompatible conducting surfaces. Cevik et al. [103] synthesized a polymer on a gold electrode using cyclic voltammetry, which was subsequently covalently linked to cytochrome C, serving as a bridge for thylakoid membrane immobilization. This resulted in a stable multilayer structure providing efficient electron transfer from thylakoid membranes to the electrode.

### 5.3. Photochemical Polymerization

Photochemical polymerization represents a promising approach for the synthesis of functional polymer materials, enabling spatiotemporal control over the process. Unlike chemical polymerization, photoinitiation ensures rapid reaction initiation at room temperature, which is particularly important for systems containing thermosensitive biological components [104,105].

The photopolymerization process begins with light absorption by either the redox-modified monomer or a photoinitiator, which transitions to an excited state followed by decomposition into active radicals that react with the monomers [104]. Subsequent chain propagation and termination steps occur analogously to those described in previous sections. Redox center attachment may also occur after polymerization.

In the context of redox polymer synthesis for biosensing, key process requirements include the creation of functional materials with controlled structure and stable biomolecule immobilization. Systems initiated by visible light are of particular interest as they are less damaging to biological molecules.

#### 5.3.1. Photoinitiators

The literature frequently describes photochemical polymerization using LAP (lithium phenyl-2,4,6-trimethylbenzoylphosphinate), activated by visible light (λ = 405–450 nm) [105,106,107]. Ozkan et al. [105] employed this photoinitiator to synthesize three-dimensional gelatin methacrylate hydrogel scaffolds. The main advantage of this method is the rapid, mild (room temperature) fabrication of biocompatible structures with complex geometries for tissue engineering.

#### 5.3.2. Spatial Control

Photochemical polymerization enables the creation of polymer materials with precise spatial organization. Using photomasks, the distribution of redox-active groups within the polymer volume can be controlled, creating regions with different concentrations. Such structured materials, in which redox centers are distributed as ordered concentration profiles rather than randomly, provide more efficient charge transfer due to optimized migration pathways [15].

### 5.4. Summary of Synthetic Methods

**Figure 13:**
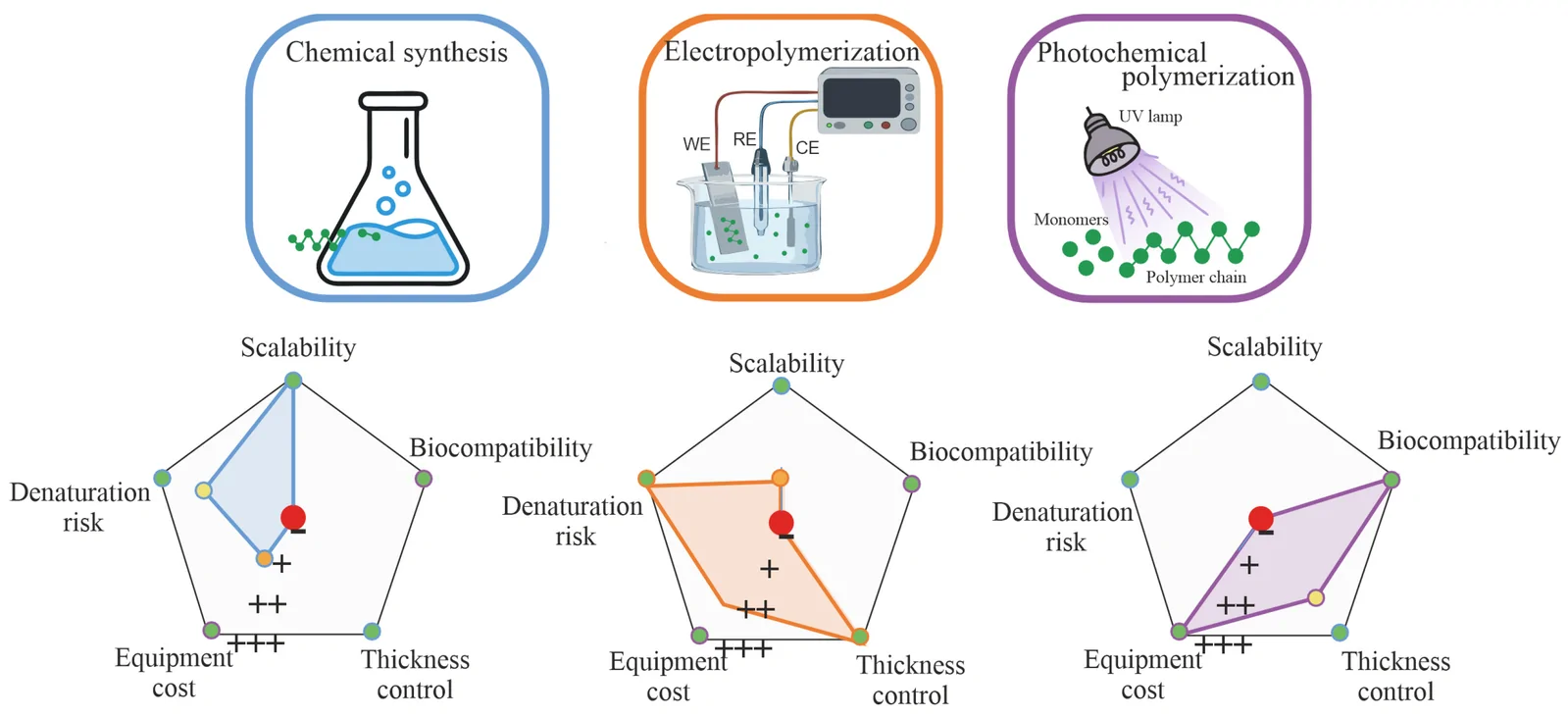
Comparative assessment of synthetic methods for redox polymers: chemical, electrochemical, and photochemical polymerization.

The literature distinguishes three main approaches to redox polymer synthesis for biosensing. Chemical synthesis is the most popular and versatile method. It provides precise control over structure and composition and is scalable but requires organic solvents and involves multiple steps, reducing its environmental friendliness. In laboratory practice and biosensor prototyping, electropolymerization is preferred. This method allows work with small volumes (50–100 µL) and precise control of film thickness but is difficult to scale and carries a risk of biomolecule denaturation. The most environmentally friendly and biocompatible approach is photochemical polymerization with visible light initiators, offering spatiotemporal control and mild conditions (room temperature and physiological environment), which is important for creating structures with living cells and implantable devices; however, this method requires more expensive equipment. Ultimately, the optimal method is determined by the task: chemical synthesis for fundamental research and bulk polymer production, electropolymerization for rapid laboratory prototypes, and photopolymerization for biomedical applications where biocompatibility and “green” chemistry are paramount. A comparative assessment of these methods is presented in Figure 13.

### 5.5. Critical Evaluation of Synthetic Approaches

While the three synthetic strategies—chemical, electrochemical, and photochemical polymerization—each offer distinct advantages, a critical evaluation reveals important practical limitations that are often understated in the literature. Chemical synthesis, despite its versatility and scalability, suffers from inherent batch-to-batch variability in molecular weight and degree of functionalization, which directly impacts the reproducibility of biosensor performance. This is particularly problematic for commercial translation, where strict quality control is essential. Electropolymerization, while elegant for rapid prototyping, is fundamentally limited by the difficulty of scaling up film deposition to large-area electrodes and by the risk of biomolecule denaturation during the polymerization process, as the applied potentials can generate reactive intermediates that damage sensitive biological receptors. Photochemical polymerization, although the most biocompatible, remains constrained by the limited penetration depth of visible light in opaque or highly absorbing solutions, restricting its application to thin films and transparent substrates. Furthermore, the photoinitiators used in these systems may generate toxic byproducts upon degradation, which is a critical concern for implantable devices. We therefore suggest that the choice of synthetic method should be guided not only by the desired film properties but also by a realistic assessment of these scalability, reproducibility, and biocompatibility constraints. Future research should prioritize the development of hybrid approaches that combine the advantages of different methods while mitigating their individual shortcomings.

## 6. Formation of Redox Polymer Layers with Immobilized Bioreceptors

**Figure 14:**
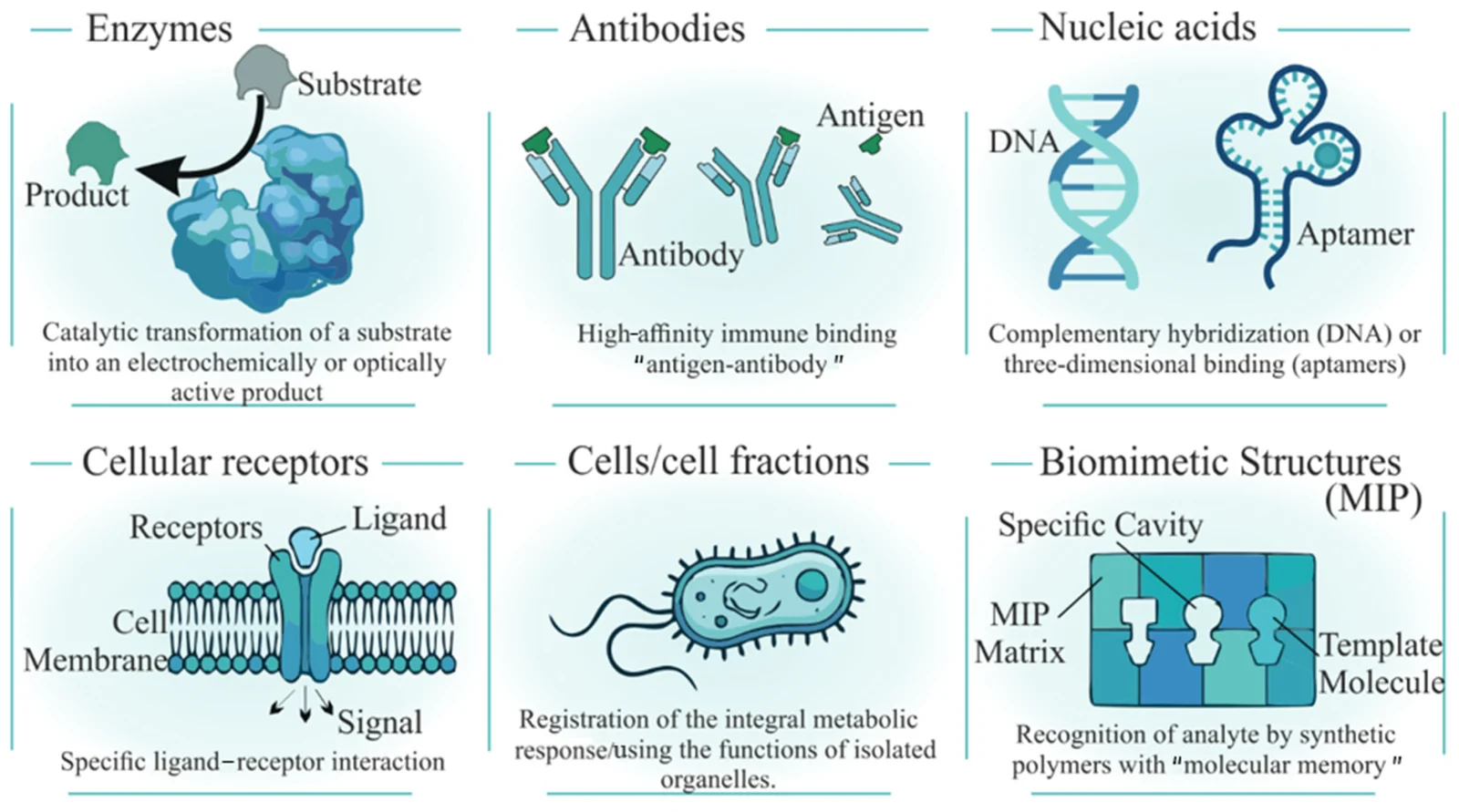
The main types of receptor elements used in biosensors based on redox polymers.

Modern biosensors employ a wide range of biological and biomimetic components for specific analyte recognition. These recognition elements are shown in Figure 14.

**Figure 15:**
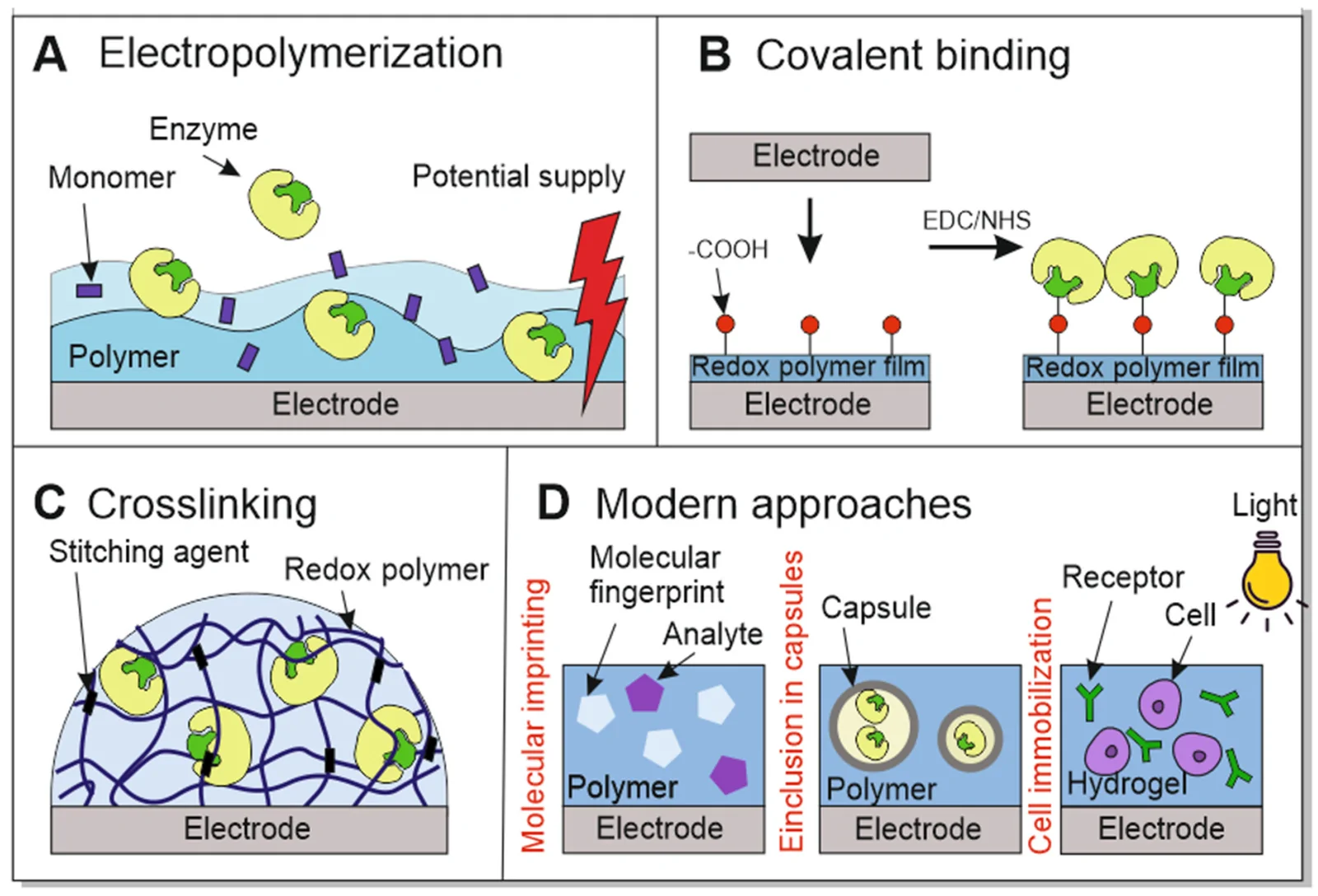
Principal strategies for biomolecule immobilization in redox polymer matrices: (**A**) physical entrapment during electropolymerization; (**B**) sequential covalent immobilization: two-step attachment of the enzyme to a pre-formed polymer film (amide bond formation and EDC/NHS); (**C**) bulk crosslinking (drop-casting method); (**D**) advanced approaches: molecular imprinting (MIP) for selective recognition; enzyme encapsulation in microcapsules; soft photopolymerization for living cell immobilization.

The fundamental basis for biosensor fabrication using redox polymers is the specific interaction between the polymer matrix and biological macromolecules. The main immobilization strategies are illustrated in Figure 15.

### 6.1. Physical Entrapment

Co-electrodeposition with physical entrapment of the biomolecule is one of the simplest approaches, in which biomolecules are captured within the polymer matrix during its formation. This mechanism is commonly encountered in electropolymerization (Figure 15A), where an appropriate potential is applied to the working electrode immersed in a solution containing both receptor molecules and monomers [61,108].

It is noteworthy that at pH 7, highly positively and highly negatively charged enzymes with similar molecular masses—such as galactose oxidase (isoelectric point pH 12) and choline oxidase (isoelectric point pH 4.5)—are immobilized with comparable efficiency [108]. These observations demonstrate that the electrochemical immobilization procedure is broadly applicable to biomolecules regardless of their isoelectric point. The main disadvantage is the potential for enzyme denaturation under the harsh conditions of polymerization and uncontrolled distribution of the biomolecule within the film.

### 6.2. Sequential Covalent Immobilization

Sequential immobilization with covalent binding of the biomolecule (Figure 15B) provides higher stability due to the formation of chemical bonds between functional groups of the polymer and the biomolecule [108]. In this approach, a pristine redox polymer film is first formed on the electrode (via electropolymerization or casting from a pre-synthesized polymer), after which the biomolecule is immobilized onto the modified electrode through covalent crosslinking. For example, in systems based on redox-center-modified polyethylenimine, amino groups of the polymer form amide bonds with carboxyl groups of enzymes (e.g., Gox and lactate dehydrogenase), preserving enzyme active site accessibility while enabling electron transport [21,58,59,73,109].

Coordinative interactions are also characteristic of this approach and are particularly effective for metal-containing polymers, where histidine fragments of recombinant proteins or natural metal-binding centers of enzymes form complexes with metal ions within the polymer matrix [110,111].

### 6.3. Bulk Crosslinking (Drop Casting)

An alternative approach involves applying an aqueous solution containing the redox polymer, biomolecule, and crosslinking agent directly onto the electrode (Figure 15C). Upon drying, chemical crosslinking of the polymer chains occurs, resulting in the biomolecule being firmly entrapped within a three-dimensional conductive network. This structure is ideally suited for operation in physiological fluids, as it mimics the natural environment of the enzyme [108,112,113].

### 6.4. Advanced Immobilization Strategies

Contemporary approaches (Figure 15D) include molecular imprinting, which creates specific cavities within the polymer that are complementary in shape and functional groups to the target biomolecule, thereby achieving selective recognition [41]. Another strategy involves the encapsulation of enzymes within microcapsules and microtubes. Several studies have examined the chemical and electrochemical preparation of polymer microcapsules, for example, using polypyrrole [108]. Such capsules have been loaded with catalase, GOx, or alcohol dehydrogenase.

For living cell immobilization, mild in situ methods are preferred, such as visible-light photopolymerization or enzymatic crosslinking, which proceed under physiological conditions (neutral pH, room temperature, and aqueous medium) and minimize cellular stress. Upon light irradiation, radical polymerization is initiated, resulting in the formation of a stable hydrogel around the cells [114,115].

After matrix formation, cells become physically entrapped within its pores. This is the primary retention mechanism, preventing cell washout by fluid flow. Peptide sequences that are recognized by integrin receptors on the cell surface are often incorporated into the polymer network, leading to the formation of focal adhesions [116].

### 6.5. Critical Comparison of Immobilization Strategies

The immobilization strategies discussed above—physical entrapment, covalent binding, and bulk crosslinking—are not interchangeable; their selection profoundly affects biosensor performance, yet this choice is often made empirically rather than rationally. Physical entrapment during electropolymerization is the simplest and most cost-effective approach, but it offers the least control over enzyme orientation and loading, leading to significant variability in sensor-to-sensor performance. Moreover, the harsh electrochemical conditions required for polymer film formation can partially denature the enzyme, reducing its specific activity and compromising the sensitivity of the final device. Covalent immobilization, while providing superior stability and controlled enzyme attachment, requires additional synthetic steps and can introduce steric hindrance that restricts substrate access to the active site, particularly for enzymes with deeply buried catalytic centers. Bulk crosslinking with agents such as glutaraldehyde, although widely used, presents a major biocompatibility concern: residual crosslinker can leach into the analyte solution, causing enzyme inactivation over time and potentially interfering with biological samples. Advanced strategies such as molecular imprinting and microencapsulation offer exciting possibilities for enhanced selectivity and stability, but their complexity and cost remain prohibitive for routine large-scale manufacturing. A critical gap in the current literature is the lack of systematic comparative studies that evaluate these immobilization strategies under identical conditions using standardized performance metrics. We advocate for future research to address this gap by providing direct, side-by-side comparisons of different immobilization methods on the same redox polymer platform, thereby enabling evidence-based selection of the most appropriate strategy for a given application.

## 7. Composite Materials for Bioreceptor Immobilization

The incorporation of composite materials into redox polymer-based biosensors represents a powerful strategy for substantially improving their analytical performance. Hybrid materials formed by introducing CNT, graphene, metal nanoparticles, and other functional components into the redox polymer matrix yield composites with enhanced properties [9].

### 7.1. Carbon Nanotubes

CNTs play a key role in increasing the electronic conductivity of the composite. When incorporated into the polymer, CNTs create a branched conductive network that facilitates charge transfer between the enzyme active site and the electrode surface [117], leading to a significant increase in amperometric response.

In another study, Lavrova et al. [56] examined the combination of single-walled carbon nanotubes (SWCNTs) with the BSA-Fc redox polymer. This nanocomposite approach effectively increases the conductive surface area, leading to improved biosensor performance. Comparative evaluation of various biological recognition elements—tyrosinase, laccase, and a membrane fraction of Delftia tsuruhatensis—demonstrated that the tyrosinase/BSA-Fc-SWCNT system exhibited the best analytical characteristics, including an impressively low detection limit for phenol of 1 × 10^−3^ mg/L [56]. In a related study, Kuznetsova and coauthors developed nanocomposite materials based on conductive polymers for glucose biosensors [118]. The researchers compared three electropolymerized mediators—neutral red, thionine, and aniline—as part of hybrid nanocomposite systems. The optimal system, based on poly(neutral red) and thermally expanded graphite, achieved a sensitivity of 1000 ± 200 nA·dm^3^/mmol and a detection limit of 0.006 mmol/L. Validation against human blood samples confirmed excellent agreement with standard methods (R^2^ = 0.9828), positioning this biosensor as a promising candidate for clinical applications. Kamanina et al. [119] developed a glucose biosensor based on a screen-printed electrode modified with a silicone sol–gel conducting matrix containing CNTs. The biosensor exhibited a linear range of 0.045–1.04 mM and was successfully applied to glucose detection in blood samples.

**Figure 16:**
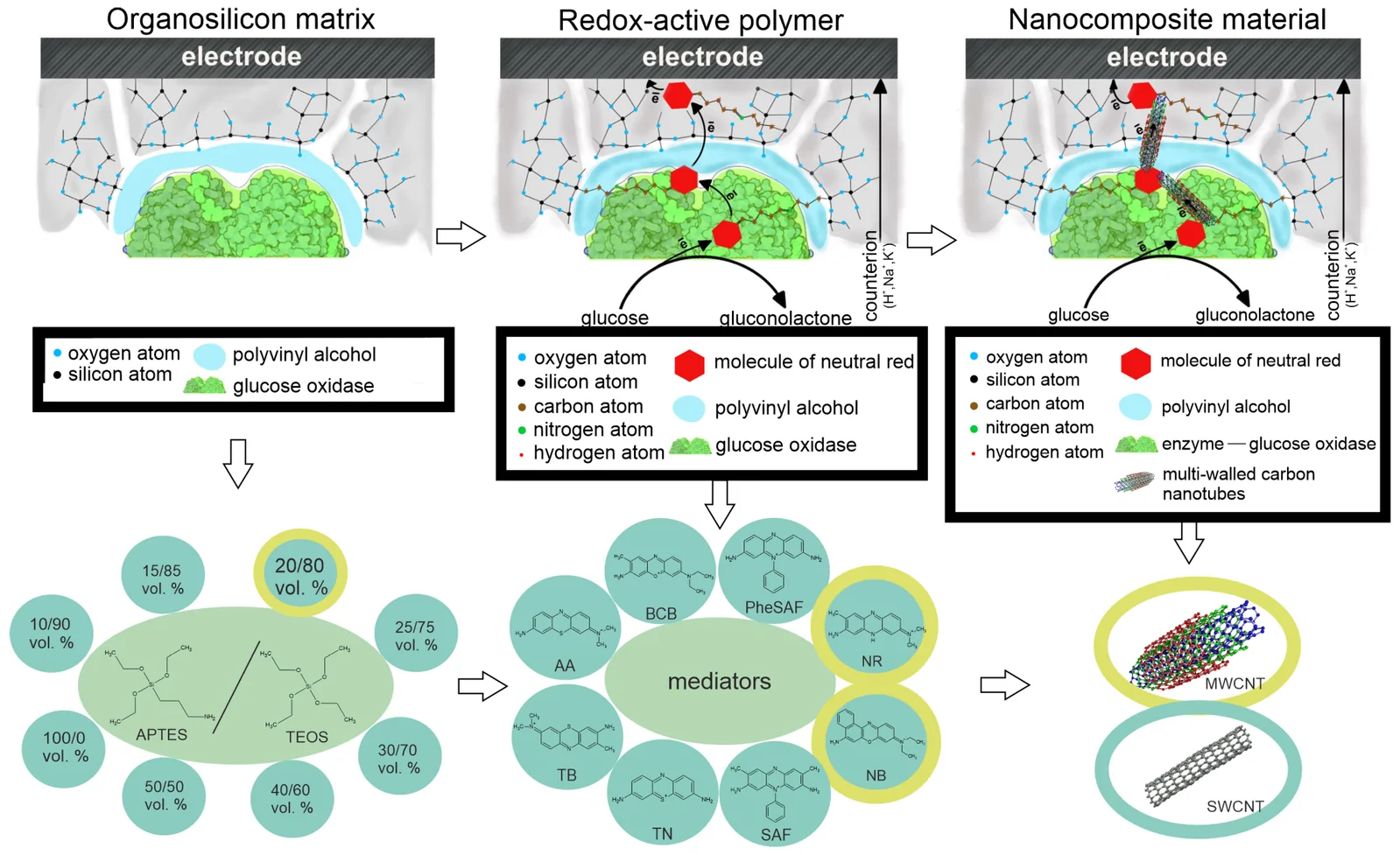
Schematic representation of the formation of the organosilicon matrix/enzyme/CNT nanocomposite material and the charge transfer mechanism in the glucose biosensor. Reproduced with permission from ref. [120]. Copyright © 2024, American Chemical Society.

An integrated approach to creating such materials was recently demonstrated in an article [120] that combined both SWCNTS and MWCNTs into a hybrid organosilicon matrix (Figure 16). The study showed that the addition of CNTs to a matrix consisting of (3-aminopropyl)triethoxysilane/tetraethyl orthosilicate and mediators (such as neutral red) creates a reliable three-dimensional conductive network. This architecture promotes rapid electron transfer and provides a stable environment for GOx, significantly increasing the sensitivity of the glucose biosensor.

### 7.2. Graphene and Its Derivatives

The application of graphene in redox polymer-based biosensors stems from its unique two-dimensional structure, which combines record-high specific surface area with high electron mobility [121,122]. Graphene oxide, rich in oxygen-containing groups (-COOH and -OH), serves as an ideal platform for covalent attachment of redox-active polymers (e.g., based on osmium complexes or quinones) and biomolecules. Upon electrochemical reduction of graphene oxide to conductive reduced graphene oxide, a hybrid material is formed that provides rapid electrical contact with the electrode. These properties enable ultra-sensitive detection of analytes such as glucose [121], H_2_O_2_ [123], and deoxyribonucleic acid [124]. Reduced graphene oxide-based sensors incorporating redox polymers exhibit low detection limits due to the high adsorption capacity of graphene and the fast charge transfer kinetics of the polymer.

Graphene Quantum Dots (GQDs). As a graphene derivative, due to edge effects, they lead to unique electronic properties, an increase in the surface area-to-mass ratio, and high conductivity; they are also easily dispersed in water and are hydrophilic [125,126]. When redox-active polymers such as PEDOT:PSS or polypyrrole and GQDs are introduced into the matrices, they act as effective anionic dopants and increase effective coupling, facilitating electron transfer between the active center of the enzyme and the electrode. Lim et al. showed that doping of PEDOT with GQD anions leads to increased delocalization of π electrons and increased interchain interaction [125]. The functional groups of GQDs (-COOH and -OH) ensure effective immobilization of enzymes through hydrogen bonds and π–π interactions, as demonstrated by Nashruddin et al. when creating a biosensor based on PEDOT:PSS/TI_3_c_2_/GQDs for glucose detection [127]. In this study, the GQD modification made it possible to increase the electrochemically active surface by two times compared to pure PEDOT:PSS (0.026 versus 0.013 cm^2^) and achieve a sensitivity of 21.64 µA·mM·cm^2^ with a detection limit of 65 microns. Thus, GQDs act as an active functional component, which makes it possible to achieve a synergistic enhancement of the electrochemical response in enzyme biosensors.

### 7.3. Fullerenes

Fullerenes (C_60_ and C_70_) in combination with conducting polymers are either covalently attached or uniformly dispersed within the polymer matrix, creating a three-dimensional network for electron transfer [128]. Such composites are particularly effective for detecting analytes requiring multi-electron transfer (e.g., NO and O_2_^−^), as fullerenes facilitate these reactions by lowering the detection potential and enhancing selectivity. Polymer–fullerene hybrid biosensors find applications in early diagnostics, for instance, in detecting oxidative stress markers.

### 7.4. Metal Nanoparticles

Metal nanoparticles, especially precious metals such as gold (Au) or platinum (Pt), are often used to improve conductivity in redox-active polymers. For example, platinum nanoparticles dispersed in a polyaniline matrix not only improve conductivity but also act as highly efficient catalysts for hydrogen peroxide oxidation—a product of the GOx enzymatic reaction—thereby enabling the construction of highly sensitive biosensors [129]. In immunosensors, gold nanoparticles often serve as a universal platform for robust attachment of bioreceptor molecules (e.g., antibodies) through the formation of stable Au–S bonds while simultaneously amplifying the electrochemical signal [130].

However, the long-term stability of nanoparticle-modified electrodes may be impaired due to Ostwald maturation, a process in which small nanoparticles dissolve and re-precipitate onto larger ones to minimize surface energy, resulting in a gradual loss of electroactive surface and catalytic activity. To overcome this limitation, recent strategies focus on core–shell nanostructures, in which a protective shell (such as silicon dioxide or a conductive polymer) insulates the metal core, preventing atomic migration and aggregation while maintaining efficient electron transfer [131,132,133]. It is also common to use monatomic catalysts (SAC) in which isolated metal atoms are stabilized on a substrate (for example, nitrogen-doped carbon or MXene), which eliminates the aggregation of nanoparticles [134].

### 7.5. Ternary Hybrid Systems

A particularly promising direction is the creation of ternary component systems. Composites combining a redox polymer, carbon nanomaterials, and noble metal nanoparticles exhibit synergistic effects [130,135,136]. Such hybrid materials enable the fabrication of highly sensitive and stable biosensor platforms for clinical diagnostics and environmental monitoring. For instance, Wu et al. [136] developed a deoxyribonucleic acid biosensor based on a glassy carbon electrode modified with fullerene (C_60_)/PAMAM dendrimer/gold nanoparticles, achieving a wide linear range (10 fM–10 nM).

### 7.6. Nanoparticle Dispersion and Electrode Modification

The preparation of stable nanoparticle suspensions for electrode modification requires proper dispersion and stabilization of the material in a suitable carrier liquid. In practice, nanomaterial powder (e.g., 1–5 mg of MWCNTs or graphene oxide) is introduced into a dispersion medium, which may be water, organic solvents (dimethylformamide and isopropanol), or aqueous solutions of surfactants such as sodium dodecyl sulfate [15,120]. To disrupt aggregates and obtain a homogeneous dispersion, the suspension is subjected to prolonged ultrasonication (30 min to several hours) using an ultrasonic bath or, more effectively, a probe-type ultrasonic disperser [56].

To stabilize the suspension and prevent re-aggregation, covalent or non-covalent functionalization of nanoparticles is often employed. For example, oxidation of CNTs with a mixture of concentrated acids introduces carboxyl (-COOH) and hydroxyl (-OH) groups on their surface, improving wettability and dispersibility in water while creating active sites for subsequent covalent binding to polymers or biomolecules [15,56].

The resulting suspension can then be deposited onto the electrode surface by drop casting, spray coating, or electrophoretic deposition. Importantly, the dispersion process and suspension composition directly affect the morphology and reproducibility of the final modified layer, determining key biosensor parameters such as working surface area, charge transfer resistance, and enzyme immobilization efficiency [15].

### 7.7. Comparative Summary and Selection Criteria

While all classes of nanomaterials demonstrably improve the analytical performance of redox polymer-based biosensors, a critical comparative analysis reveals that their effectiveness is highly context-dependent. The selection of a nanomaterial should be guided by the specific bottleneck limiting sensor performance rather than by a general assumption of “better conductivity.”

Carbon nanotubes (CNTs) are most effective when the primary limitation is slow heterogeneous electron transfer at the electrode–polymer interface. Their high aspect ratio and excellent intrinsic conductivity create a percolating network that substantially lowers charge transfer resistance. This is particularly beneficial for oxidases with deeply buried active sites, where efficient “wiring” depends on maximizing the number of redox centers in close proximity to a conductive pathway. However, the performance enhancement is highly sensitive to CNT dispersion quality: aggregated nanotubes not only fail to improve conductivity but can also block access to the polymer matrix, reducing effective enzyme loading.

Graphene and its derivatives excel in applications where high bioreceptor loading and stable immobilization are paramount. The large specific surface area of graphene provides numerous anchoring sites for both redox polymers and enzymes, while its oxygen-containing functional groups (in graphene oxide) enable facile covalent conjugation. The key unresolved challenge, however, is the irreversible agglomeration of graphene sheets during electrode modification, which significantly reduces its effective surface area. Moreover, the electrochemical reduction of graphene oxide to reduced graphene oxide—necessary to restore conductivity—is often incomplete, leading to residual defects that can act as recombination centers for charge carriers.

Metal nanoparticles (Au and Pt) serve a fundamentally different role: they provide catalytic signal amplification by directly catalyzing the electrooxidation or electroreduction of enzymatic reaction products (e.g., H_2_O_2_ in oxidase-based sensors). This dual function—enhancing conductivity and providing electrocatalytic activity—makes them particularly valuable in oxidase-based sensors where product detection is a limiting step. However, their high surface energy makes them prone to Ostwald ripening and aggregation over time, leading to gradual loss of catalytic activity and sensor signal drift.

Critically, the most pronounced synergistic effects are observed in ternary hybrid systems (redox polymer + carbon nanomaterial + metal nanoparticles). In such architectures, each component addresses a distinct limitation: the polymer provides a biocompatible matrix for enzyme immobilization and mediates electron transfer; the carbon nanomaterial establishes a high-surface-area conductive network; and the metal nanoparticles catalyze the final signal-generating reaction. This tripartite synergy enables performance metrics—particularly sensitivity and limit of detection—that are unattainable with any binary combination. For instance, the combination of SWCNTs with BSA-Fc and tyrosinase achieved an exceptionally low detection limit for phenol of 1 × 10^−3^ mg/L [56], while glucose biosensors incorporating poly(neutral red) with thermally expanded graphite reached sensitivities of 1000 ± 200 nA·dm^3^/mmol and a detection limit of 0.006 mmol/L [118].

From a practical and translational perspective, however, these composites face significant obstacles. The complexity of multi-component formulations makes quality control and batch-to-batch reproducibility extremely challenging, representing a major barrier to commercial scale-up. Furthermore, the long-term stability of these nanomaterials in biological fluids remains poorly characterized; issues such as nanoparticle leaching, protein corona formation on carbon surfaces, and gradual detachment of enzyme from the composite layer can compromise sensor performance over extended operation periods. We therefore propose that future research should prioritize not only the development of new composite formulations but also systematic studies of their degradation mechanisms and shelf-life under real-world operating conditions.

## 8. Redox Polymer-Based Biosensors and Their Nanocomposites

The fabrication of a functional biosensor element is a multi-stage process aimed at forming a stable, conductive, and biocompatible layer on the electrode surface. This process follows a defined sequence of steps based on the synthetic and modification methods described in previous sections.

### 8.1. Stepwise Fabrication Protocol

Step 1: Electrode surface preparation. This key step ensures the adhesion of subsequent layers. It involves mechanical polishing with abrasive pastes (e.g., diamond or aluminum oxide), ultrasonic cleaning in solvents (acetone, ethanol, and water), and electrochemical activation in acidic or alkaline media by potential cycling. These procedures yield a reproducible, clean, and activated surface with a maximal density of functional groups.

Step 2: Electrode modification with nanomaterials. Deposition of a nanomaterial layer increases the conductive surface area and enhances the signal.

Step 3: Formation of the redox polymer layer with immobilized bioreceptor. This central step determines the operational characteristics of the sensor. The layer is formed via co-deposition with the biomolecule or sequential modification with covalent crosslinking. It can be deposited either onto a pre-formed nanomaterial layer or via co-precipitation with nanoparticles in a single composite.

Step 4: Application of an external selective membrane layer (if required). To enhance selectivity in complex matrices (blood and serum) and prevent electrode fouling, selective membranes are applied over the biosensitive layer. These may include Nafion-type polymers (which repel anions such as ascorbic acid) or polyurethanes that regulate analyte diffusion.

### 8.2. Biosensor Operation and Regeneration

An important feature of a properly designed biosensor is its regenerability. Since the bioreceptor and mediator are not consumed but are continuously cyclically regenerated (the receptor by the mediator and the mediator by the electrode), the same biosensor can be used for multiple measurements. Long-term stability is determined by the preservation of the active enzyme conformation within the polymer and the stability of the polymer layer itself [14].

The analyte sample (e.g., a drop of blood, a food product solution, or a wastewater sample) is introduced into the cell containing an electrolyte (typically phosphate-buffered saline). The analyte diffuses to the sensor surface and initiates the previously described cascade of reactions: binding to the enzyme active site, electron transfer to the redox centers of the polymer, and their subsequent oxidation at the electrode.

After measurement, reusable laboratory sensors undergo a regeneration procedure. The electrode is rinsed with clean buffer solution to remove residual analyte and possible reaction products (e.g., hydrogen peroxide in the case of oxidases).

### 8.3. Comparative Analysis of Redox Polymer-Based Biosensors

A comparative analysis of biosensors based on different redox polymers is presented in Table 3. The table summarizes key analytical parameters including detection limit, linear range, and sensitivity for representative systems.

Table 3 shows the analytical characteristics of typical electrochemical sensors based on redox polymers, which have been reported over the past decade. The data obtained clearly demonstrate that redox polymers have evolved from classical glucose biosensors (based on Os complexes or Fc) to highly developed platforms capable of detecting a wide range of analyzed substances, including neurotransmitters (dopamine and adrenaline), hormones (oxandrolone), cancer biomarkers (r-tau 181), heavy metals (Pb ^2+^, Hg^2+^, and Cd^2+^), pesticides (glyphosate) and new pollutants (PFAS). The main trends include the transition from enzyme-based sensors to synthetic recognition elements such as molecular fingerprint polymers (MIPs), which provide increased stability and reduced cost; the integration of redox polymers with carbon nanomaterials (CNTs and graphene) and metal nanoparticles to increase sensitivity and transfer electrons; and the expansion of the scope of practical application, from clinical diagnostics to environmental monitoring and food safety. TEMPO-based polymers and conductive polymer–MIP hybrids represent the latest advances that allow for detection without the use of reagents with ultra-low detection limits up to the subnanomolar range.

### 8.4. Critical Remarks on Comparative Analysis

Despite the impressive diversity of analytes and sensing platforms summarized in Table 3, a critical examination reveals several important caveats that are often overlooked. First, the reported limits of detection (LODs) and sensitivities are rarely comparable across different studies due to significant variations in experimental conditions, including electrode geometry, measurement techniques (amperometry vs. voltammetry), and electrolyte composition. This lack of standardization complicates direct performance comparisons and underscores the urgent need for unified testing protocols in the field. Second, the vast majority of these biosensors have been validated only in buffered solutions or artificial samples, with limited data on their performance in complex real-world matrices such as whole blood, serum, or untreated wastewater. The translation from academic demonstrations to practical applications therefore remains a critical bottleneck. Third, while MIP-based sensors and TEMPO-containing polymers represent exciting advances, their long-term operational stability and resistance to fouling in continuous monitoring scenarios have not been systematically evaluated. Therefore, science needs additional research and thorough testing in real-world conditions, as well as standardized performance indicators that allow for a meaningful cross-comparison of various redox polymer systems.

## 9. Challenges, Limitations, and Mitigation Strategies

Despite significant progress, the development and practical application of biosensors based on redox polymers faces a number of serious limitations. The problems related to their origin are listed below, as well as the appropriate strategies for their elimination.

### 9.1. Metal Leaching from Coordination Complexes

The problem of redox polymers is the leaching of metal ions from metal-containing redox centers, in particular osmium and ruthenium complexes. During long-term operation, metal ions are gradually released from the polymer matrix, which leads to an irreversible loss of electrochemical activity and potential contamination of the analyzed sample.

To mitigate the effects, new chelating ligands with increased stability constants of the complex as well as crosslinking agents, which additionally fix metal centers in a polymer grid, are used [11,59].

### 9.2. Degradation of Organic Redox Centers

Organic redox centers such as TEMPO, phenazines, and viologenes are degraded by repeated potential cycling. During successive redox transformations, radical centers may undergo undesirable side reactions, including disproportionation or recombination, which reduce the overall power and sensitivity of the sensor.

To reduce this disadvantage, the introduction of bulk substituents is used to protect the radical center and embed redox groups into rigid conjugated polymer frameworks, which limits their mobility and prevents undesirable interactions [48,57].

### 9.3. Toxicity of Components

The potential toxicity of some components used in the production of redox polymers limits their use in implantable devices. For example, PEDOT:PSS, which is widely used due to its high electrical conductivity, may contain impurities and provoke inflammatory reactions upon contact with a biological environment. Similar concerns are associated with crosslinking agents such as glutaraldehyde. To solve the problem, they use the development of “green” synthetic protocols using non-toxic reagents and natural polymer matrices (chitosan, dextran, and gelatin) that are biocompatible [82,83].

### 9.4. Scalability and Reproducibility

Reproducible industrial production remains one of the most serious obstacles to commercialization, as it is associated with a number of problems. Chemical synthesis is difficult to scale because it is very sensitive to reaction parameters, and even minor batch-to-batch changes can significantly alter the electrochemical characteristics of a redox polymer. Manual drip application of redox polymers depends on the operator and allows for films with significant differences in thickness, uniformity, and adhesion to the electrode surface, which is an obstacle to regulatory approval or mass production. Using automatic dosing solves this problem but requires precise calibration. Many high-performance redox hydrogels are often synthesized using special multistep procedures that are impractical for large-scale production. The stability of storage conditions becomes critically important when switching to a commercial product. Freshly prepared polymer films are usually used in laboratory experiments, but a real biosensor must function reliably after several weeks or months of storage in environmental conditions, since redox centers can gradually collapse and hydrolyze, and the film morphology may change upon drying. Expensive precious metal precursors (in particular, osmium and ruthenium complexes), specialized solvents, and time-consuming purification and synthesis steps increase the cost of production. Current cost-reduction efforts include process automation, electrochemical deposition methods, and a gradual transition to commercially available redox hydrogels with certified technical characteristics, but these partial solutions do not solve the full range of problems listed above, and widespread commercial use of biosensors based on redox polymers will require significant advances in synthesis methodology, quality assurance protocols, and technological engineering [19,99].

### 9.5. Biofouling in Complex Biological Matrices

When working with real biological samples, proteins, cellular elements, and other macromolecules are non-specifically adsorbed on the surface, blocking redox centers and disrupting electron transfer. The most common solution to the problem is the use of external selective membranes that repel large molecules and anionic interferents while ensuring the diffusion of the analyte with a low molecular weight [19]. An additional strategy includes covalent grafting of hydrophilic polymers (e.g., polyethylene glycol) to create antifouling coatings.

## 10. Future Directions and Emerging Trends

### 10.1. Computational Design and Machine Learning

Machine learning and multiscale modeling make it possible to predict the electron diffusion coefficient, redox potential, and compatibility with the active centers of enzymes before synthesis begins. Karlsson et al. [48] demonstrated how ML models predict electrochemical properties and allow for “reverse engineering”: the selection of polymer structures according to desired parameters. More recently, Ma et al. [51] predicted the electron diffusion coefficients in polymers containing phthalimide, increasing them by three orders of magnitude compared to reference systems.

### 10.2. Biohybrid Materials

An interesting direction is the development of biohybrid materials that combine redox polymers with living cells, organelles, or natural membranes and use complex metabolic pathways to detect analytes for which there are no commercially available enzymes. Cevik et al. [103] used a redox polymer to efficiently transfer electrons from thylakoid membranes to an electrode. This has opened up prospects for biophotoelectric converters.

### 10.3. Self-Healing Materials

A relevant area for implantable devices and wearable sensors is self-healing materials. The inclusion of dynamic covalent bonds in the structure of the redox polymer ensures regeneration after mechanical damage. Dong et al. [54] developed a conductive polyurethane containing CNTs, capable of self-healing and recycling while maintaining electrochemical activity.

### 10.4. Multiplexed Sensing

Materials for the simultaneous detection of several substances are obtained using microelectrode matrices, each of which is modified with a specific redox polymer, or the development of a single composite material with several redox centers with distinguishable electrochemical characteristics. Gan et al. [142] presented a biosensor based on chitosan and modified Fc-branched PEI for simultaneous determination of glucose and lactate, key markers of postoperative complications. An example of multiplex analysis is also the work [56], which presents a four-channel biosensor for simultaneous determination of glucose, lactate, ethanol, and starch.

### 10.5. Eco-Materials

Green synthesis using natural polymers, aqueous media, non-toxic solvents, and enzymatic polymerization is an urgent task for the introduction of redox polymers in medicine. The use of natural polymers such as chitosan, dextran, alginate, and proteins as matrices ensures not only biocompatibility but also the ability to decompose. Su et al. [84] report on chitosan-based redox-sensitive polymer conjugates for targeted doxorubicin delivery with the potential for their use in theranostics. Huang et al. [77] created a deoxyribonucleic acid hydrogel containing TEMPO, which simultaneously serves as a contrast sensor for magnetic resonance imaging and a platform for the treatment of osteoarthritis, with the possibility of subsequent biodegradation.

### 10.6. Critical Assessment of Emerging Trends

Machine learning-based design and multiplex sensing are already yielding tangible results and are being actively implemented in research, simplifying and facilitating the work of researchers. On the contrary, self-healing materials and biohybrid systems with living cells face problems in reproducibility and long-term stability in real conditions. A pragmatic, step-by-step approach with a gradual increase in stability, reproducibility, and cost effectiveness may be more effective for the development of redox-active polymers and their distribution than innovations that have not yet reached technical maturity.

## 11. Conclusions

Redox polymers have taken a special place in the development of electrochemical biosensors. They effectively solve the problem of slow electron transfer from the biomaterial to the electrodes by replacing soluble mediators while being multifunctional: the same polymer serves as the basis for the immobilization of biomolecules, a stabilizing medium, and an intermediary for rapid electron transfer between redox centers, the biomaterial, and the electrode. The greatest potential is realized in hybrid composite materials, where redox polymers are combined with carbon nanomaterials and metal nanoparticles, since this combination synergistically increases electrical conductivity, increases surface area, and improves catalytic properties, which leads to the creation of biosensors with high metrological characteristics.

Redox polymers have evolved from a research material into versatile platforms of modern analytical chemistry, underlying the development of non-reactive biosensors suitable for continuous monitoring. Currently, the greatest practical effect has been demonstrated in clinical diagnostics, especially in continuous glucose monitoring (CGM) systems, wearable sensors, and implantable devices for the treatment of chronic diseases.

In order for this potential to be fully realized, it is necessary to systematically solve problems related to overcoming biofouling, ensuring the long-term stability of redox centers, integrating machine learning not only for designing materials, predicting their properties, real-time signal processing and calibration, and developing environmentally friendly (“green”) production processes but also to facilitate the transition from laboratory prototypes to commercially viable products.

## Figures and Tables

**Table 2:** Characteristics of polymer matrices used in redox polymer fabrication.

Polymer Matrix	Examples	Interaction Sites	Key Advantages/Disadvantages	Sources
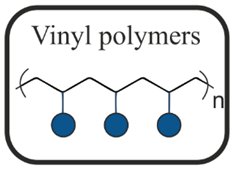	PVP and poly(vinylimidazole)	The nitrogen of the heterocycle (pyridine and imidazole) is the coordination of metals (Os and Ru)	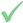 The classic base for metal complexes 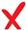 Non-specific adsorption of interfering substances	[53,58]
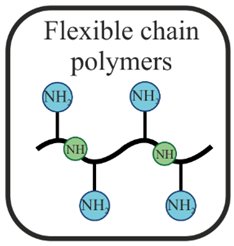	PEI and poly(allylamine)	Primary (-NH_2_) and secondary (-NH-) amines	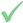 High density of redox groups and simplicity of synthesis 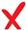 The difficulty of controlling the degree of functionalization 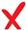 Tendency to swell/dissolve with pH changes	[21,72]
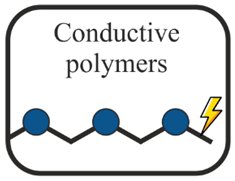	Polypyrrol (PPy), PEDOT, polythiophenes and polyphenylenes	Conjugate π-chain system; introduced side groups	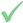 Conductivity 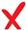 Unpredictability of properties during modification	[57,61,78,79,80]
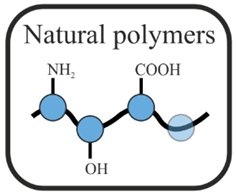	Dextrans, chitosan and BSA	Dextran: -OH; chitosan: -OH and -NH_2_; BSA: -COOH, -NH_2_, -SH, and imidazole	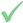 Biocompatibility and biodegradability 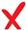 Low thermal and chemical stability	[19,56,82,83,84,85]
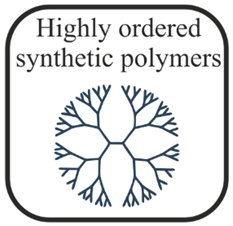	Dendrimers (PAMAM)	-NH_2_ on the surface; internal -N<; -COOH (in carboxylated forms)	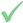 High and controlled group density 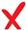 Expensive and complex synthesis	[86]

**Table 3:** Comparative analysis of biosensors based on various redox polymers.

Redox Material/Sensing Layer	Analyte	LOD	Linear Range	Sensitivity	Scope/Application	Sources
Os-hydrogel + GOx on SWCNT	Glucose	56 nM	1.0 × 10^3^–1.0 × 10^6^ nM	826.3 nA·μM^−1^·cm ^−2^	Clinical diagnostics/blood plasma	[137]
Os-polymer + macroporous PANI + GOx	Glucose	8 × 10^5^ nM	5.0 × 10^6^–6.5 × 10^7^ nM	3.54 nA·μM^−1^·cm ^−2^	Clinical diagnostics/proof-of-concept study in buffer	[138]
P(GMA-co-VFc)/MCNT	H_2_O_2_	0.34 nM	6–108 nM	0.01671 nA·μM^−1^	Clinical, environmental, and forensic analysis proof-of-concept study in buffer	[139]
P(GMA-co-VFc)	H_2_O_2_	0.66 nM	6–156 nM	0.00129 nA·μM^−1^	Clinical, environmental, and forensic analysis proof-of-concept study in buffer	[139]
Poly(acridine orange)/graphene	Uric acid	20 nM	100–1.0 × 10^6^ nM	–	Clinical diagnostics/human blood serum and human urine	[140]
Poly(sunset yellow)	Dopamine	900 nM	2.0 × 10^4^–7.0 × 10^4^ nM	–	Clinical diagnostics/proof-of-concept study in buffer	[141]
CHIT-Fc-co-BPEI-Fc + GOx	Glucose	4.7 × 10^4^ nM	1.0 × 10^5^–2.0 × 10^6^ nM	2.89 nA·μM^−1^	Clinical diagnostics/postoperative flap monitoring	[142]
CHIT-Fc-co-BPEI-Fc + lactate oxidase	Lactate	1.72 × 10^5^ nM	5.0 × 10^5^–1.5 × 10^6^ nM	2.95 nA·μM^−1^	Clinical diagnostics/postoperative flap monitoring	[142]
Poly(Alizarin Red S)/pyrolytic graphite	Oxandrolone	0.49 nM	9.8–196 nM	–	Clinical diagnostics/artificial urine	[143]
Poly(Evans Blue)/AGCE	Hydroquinone	130 nM	1.0 × 10^3^–1.0 × 10^5^ nM	1010 nA·μM^−1^·cm ^−2^	Environmental monitoring/tap and river/lake water	[144]
Poly(Evans Blue)/AGCE	Catechol	120 nM	1.0 × 10^3^–1.0 × 10^5^ nM	950 nA·μM^−1^·cm ^−2^	Environmental monitoring/tap and river/lake water	[144]
BSA-Ferrocenecarboxaldehyde/SWCNT + B. adeninivorans yeast	BOD	–	1.5–19 mg/cm^3^	–	Environmental monitoring/surface water	[145]
Polyphenol Red (pPhR) MIP	p-tau 181 (Alzheimer’s)	0.98 pg mL^−1^	–	4.39 µA·mL·pg ^−1^	Clinical diagnostics/plasma and blood serum (Alzheimer’s diagnosis)	[40]
Poly(diaminonaphthalene)/Remazol blue R (OPDAN/RBR)	Adrenaline	37 nM	1.0 × 10^3^–1.5 × 10^5^ nM	2.6 × 10^3^ nA·µM^−1^	Clinical diagnostics/blood and urea (simultaneous with paracetamol)	[146]
Polydiaminonaphthalene/Remazol blue R (OPDAN/RBR)	Paracetamol	25 nM	1.0 × 10^3^–1.5 × 10^5^ nM	3.5 × 10^3^ nA·µM^−1^	Clinical diagnostics/blood and urea (simultaneous with adrenaline)	[146]
PEDOT-TEMPO-MIP	Perfluorooctanoic acid	6.8 × 10^−4^ nM	1.0 × 10^−2^–1.0 × 10^3^ nM	–	Environmental monitoring/drinking water and surface water	[147]
TEMPO-post-functionalized ROMP polymer	Staphylococcus aureus	2.2 CFU/mL	10^1^–10^8^ CFU/mL	–	Food safety/milk and orange juice	[148]
Poly(2-(azulen-1-yldiazenyl)-5-(thiophen-2-yl)-1,3,4-thiadiazole)/GCE Hg(II)	Hg(II)	200 nM	–	–	Environmental monitoring/water pollution	[149]
Poly(copper(II) porphyrin)	Glyphosate	1000 nM	2.0 × 10^3^–1.2 × 10^5^ nM	–	Environmental monitoring/rainwater	[150]
Fc-poly-L-lysine+GOx	Glucose	23 × 10^3^ nM	0–1.0 × 10^7^ nM	6.55 nA·cm^−2^·µM^−1^	Clinical diagnostics/laboratory model glucose monitoring	[151]
Os redox polymer + GOx-grafted MWCNT	Glucose	–	–	3.18 × 10^3^ ± 0.30 × 10^3^ nA·μM^−1^·cm ^−2^	Clinical diagnostics/laboratory model glucose monitoring	[152]
Os-polymer/GOx hydrogel in microfluidic channel	Glucose	5 × 10^5^ nM	1.0 × 10^7^ nM	4.69 nA·μM^−1^· cm ^−2^	Cell culture monitoring/in vitro culture media analysis	[45]
Ferrocene-labeled e-NanoMIP	Glucose	4.3 × 10^5^ nM	8.0 × 10^5^–5.0 × 10^7^ nM	(5.57 × 10^3^ ± 0.79 × 10^3^) nA·μM^−1^	Clinical, environmental, and forensic analysis/proof-of-concept study in buffer	[153]
Ferrocene-labeled e-NanoMIP	Paracetamol	8.2 × 10^4^ nM	1.0 × 10^5^–1.0 × 10^6^ nM	(1.01 × 10^4^ ± 0.27 × 10^3^) nA·μM^−1^	Clinical, environmental, and forensic analysis/proof-of-concept study in buffer	[153]
Ferrocene-labeled e-NanoMIP	C4-homoserine lactone	0.12 nM	6.25 × 10^3^–8.0 × 10^5^ nM	(4.20 × 10^7^ ± 1.73 × 10^6^) nA·μM^−1^	Clinical, environmental, and forensic analysis/proof-of-concept study in buffer	[153]
Ferrocene-labeled e-NanoMIP	Tetrahydrocannabinol	0.05 nM	100–1.0 × 10^6^ nM	(7.20 × 10^6^ ± 0.45 × 10^6^) nA·μM^−1^	Clinical, environmental, and forensic analysis/proof-of-concept study in buffer	[153]
Ferrocene-labeled e-NanoMIP	Trypsin	0.2 nM	6.5–100 nM	2.50 × 10^5^ nA·μM^−1^	Clinical, environmental, and forensic analysis/proof-of-concept study in buffer	[153]
PEDOT/ Alg/GNP/LOx-h	L-lactate	4 × 10^5^ nM (<5 × 10^5^ nM); 3.5 × 10^5^ nM (>5 × 10^5^ nM)	<5.0 × 10^6^–1.0 × 10^8^ nM	0.0216 nA·μM^−1^·cm ^−2^	Clinical diagnostics/Proof-of-concept study in buffer	[154]
Poly(acrilyc acid):PEDOT-Prussian Blue + LOx	Lactate	1.45 × 10^3^ nM	5.0 × 10^3^–4.0 × 10^6^ nM	223 ± 3 nA·μM^−1^·cm ^−2^	Clinical diagnostics/artificial sweat	[155]
3D porous PEDOT/AuNP + MB indicator	miRNA24	3.8 × 10^−7^ nM	1.0 × 10^−6^–10 nM	–	Clinical diagnostics/clinical microRNA biomarker detection	[156]
P.yeei/CHIT-FC-CNT	BOD	–	0.1–4.5 mg/cm^3^	–	Environmental monitoring/surface water	[44]
Au/CP/Mercaptohenaxol/Apt-Fc	Cd^2+^	1.644 × 10^−2^ nM	0.1–1000 nM	–	Food analysis/orange and lettuce	[157]
Au/CP/Mercaptohenaxol/Apt-Methylene Blue	Pb^2+^	8.931 × 10^−2^ nM	0.1–1000 nM	–	Food analysis/orange and lettuce	[157]
Polypyrrole	Pb	70 nM	150–8.0 × 10^5^ nM	–	Environmental monitoring/proof-of-concept study in buffer	[158]
Cr(III)-Imprinted polymer	Cr	17.6 nM	100–1.0 × 10^4^ nM	4.25 × 10^3^ nA·μM^−1^	Environmental monitoring and clinical diagnostics/tap, river water, and urine	[159]
Laccase/poly-anthranilic acid	p-nonylphenol	1.74 nM	5–250 nM	–	Environmental monitoring/tap and lake water	[160]
Cathode: Methylene blue/Tris(2-carboxyethyl)phosphine/Polydopamine-Fe_3_O_4_ Anode: Ru(bpy)_3_^2+^/Tripropylamine/Boron nitride quantum dots	*Pseudomonas aeruginosa*	Visual ECL: 0.34 CFU/mL Photothermal: 0.40 CFU/mL	0–10^7^ CFU/mL	–	Food analysis/chicken breast and milk	[161]
PAMAM/poly(neutral red)	L-dopa	26 nM	80–2.4 × 10^6^ nM	–	Food and drug safety/Syndopa tablets	[162]
PAMAM/poly(neutral red)	H_2_O_2_	1.5 × 10^3^ nM	4.6 × 10^3^–2.2 × 10^6^ nM	–	Food and drug safety/juices	[162]
Carbon black/pillar [5]arene/poly(neutral red)	Doxorubicin	3 nM	10–1.0 × 10^5^ nM	–	Clinical diagnostics/synthetic blood plasma	[163]
Poly(thionine)-nanogold nanocomposite	Trypsin	2.5 × 10^−2^ nM	4.3 × 10^−2^–8.6 nM	–	Clinical diagnostics/human serum of pancreatic disease patients	[164]

## Data Availability

No new data were created or analyzed in this study. Data sharing is not applicable to this article.

## Abbreviations

The following abbreviations are used in this manuscript:

BSA	Bovine Serum Albumin
CNTs	Carbon Nanotubes
EDC	1-Ethyl-3-(3-Dimethylaminopropyl)carbodiimide
Fc	Ferrocene
GOx	Glucose Oxidase
GQDs	Graphene Quantum Dots
LOD	Limit Of Detection
MIP	Molecularly Imprinted Polymer
MWCNTs	Multi-Walled Carbon Nanotubes
NHS	N-Hydroxysuccinimide
PAMAM	Poly(amidoamine)
PEDOT	Poly(3,4-ethylenedioxythiophene)
PEG	Polyethylene Glycol
PEI	Polyethylenimine
PVP	Poly(vinylpyridine)
SWCNTs	Single-Walled Carbon Nanotubes
TEMPO	2,2,6,6-Tetramethylpiperidine-1-Oxyl
